# A Nitric Oxide-Responsive Transcriptional Regulator NsrR Cooperates With Lrp and CRP to Tightly Control the *hmpA* Gene in *Vibrio vulnificus*

**DOI:** 10.3389/fmicb.2021.681196

**Published:** 2021-05-21

**Authors:** Garam Choi, Dukyun Kim, Hanhyeok Im, Sang Ho Choi

**Affiliations:** ^1^National Research Laboratory of Molecular Microbiology and Toxicology, Department of Agricultural Biotechnology, Seoul National University, Seoul, South Korea; ^2^Center for Food and Bioconvergence, Seoul National University, Seoul, South Korea

**Keywords:** *Vibrio vulnificus*, gene regulation, transcriptional regulator, nitric oxide dioxygenase, nitric oxide, nitrosative stress, stress response

## Abstract

Nitric oxide (NO) is an important antimicrobial effector produced by the host innate immune system to counteract invading pathogens. To survive and establish a successful infection, a fulminating human pathogen *Vibrio vulnificus* expresses the *hmpA* gene encoding an NO dioxygenase in an NO-responsive manner. In this study, we identified an Rrf2-family transcriptional regulator NsrR that is predicted to contain the Fe-S cluster coordinated by three cysteine residues. Transcriptome analysis showed that NsrR controls the expression of multiple genes potentially involved in nitrosative stress responses. Particularly, NsrR acts as a strong repressor of *hmpA* transcription and relieves the repression of *hmpA* upon exposure to NO. Notably, *nsrR* and *hmpA* are transcribed divergently, and their promoter regions overlap with each other. Molecular biological analyses revealed that NsrR directly binds to this overlapping promoter region, which is alleviated by loss of the Fe-S cluster, leading to the subsequent derepression of *hmpA* under nitrosative stress. We further found that a leucine-responsive regulatory protein (Lrp) negatively regulates *hmpA* in an NsrR-dependent manner by directly binding to the promoter region, presumably resulting in a DNA conformation change to support the repression by NsrR. Meanwhile, a cyclic AMP receptor protein (CRP) positively regulates *hmpA* probably through repression of *nsrR* and *lrp* by directly binding to each promoter region in a sequential cascade. Altogether, this collaborative regulation of NsrR along with Lrp and CRP enables an elaborate control of *hmpA* transcription, contributing to survival under host-derived nitrosative stress and thereby the pathogenesis of *V. vulnificus*.

## Introduction

Nitric oxide (NO) is a highly reactive, toxic, and membrane-permeable radical gas. As one of the major components of the host innate immune system, NO is produced by inducible NO synthase (iNOS) which is expressed in phagocytes and epithelial cells under infectious conditions (Fang, 2004; Wang et al., 2010). NO produced by iNOS can subsequently be converted into other toxic reactive nitrogen species (RNS) such as nitrogen dioxide (NO_2_), peroxynitrite (ONOO^−^), and dinitrogen trioxide (N_2_O_3_) which impose the nitrosative stress on pathogens (Fang, 2004; Stern and Zhu, 2014). Furthermore, intestinal commensals can reduce nitrate (${\text{NO}}_{3}^{-}$) in the diet to nitrite (${\text{NO}}_{2}^{-}$), which interacts with gastric acid, resulting in RNS that act as antimicrobial barriers against ingested enteric pathogens (Sobko et al., 2005; Tiso and Schechter, 2015). RNS can cause damage to cellular components, including the metal centers of proteins, membrane lipids and nucleotide bases, and thereby inhibit respiration and interfere with the DNA replication of pathogens (Fang, 2004). Therefore, pathogens have evolved sophisticated mechanisms to sense the increased level of RNS and express the proper genes to overcome nitrosative stress in a host (Bang et al., 2006; Stern et al., 2012).

To understand the NO-responsive gene expression in pathogens, numerous transcriptional regulators have been characterized (Spiro, 2007). Among them, two transcriptional regulators, NorR and NsrR, are known to have focused functions on sensing NO in a wide range of bacteria (Stern and Zhu, 2014). NorR directly recognizes NO using its non-heme iron center and controls the expression of genes for NO detoxification: *norVW* in *Escherichia coli* and *hmpA* and *nnrS* in *Vibrio cholerae* (D'Autreaux et al., 2005; Stern et al., 2012). Meanwhile, NsrR uses an iron-sulfur (Fe-S) cluster as a cofactor to directly sense NO and regulates a variety of genes involved in NO detoxification and NO damage repair, particularly *hmpA* in *E. coli, Salmonella enterica* serovar Typhimurium, and *Streptomyces coelicolor* (Bang et al., 2006; Spiro, 2007; Tucker et al., 2008). The NO-responsive Fe-S cluster is coordinated to NsrR by three cysteine residues which are widely conserved in various bacterial NsrR (Tucker et al., 2010). A recent study showed that both [2Fe-2S] and [4Fe-4S] clusters can be coordinated to *S. coelicolor* NsrR (Crack et al., 2015). Upon exposure to NO, the Fe-S cluster is nitrosylated, forming the iron-nitrosyl species such as dinitrosyl iron complex (DNIC), Roussin's Red Ester (RRE), and Roussin's Black Salt (RBS) (Serrano et al., 2016; Crack and Le Brun, 2019). The resulting apo-NsrR lacking an intact Fe-S cluster shows a distinct protein conformation from that of holo-NsrR, leading to loss of DNA-binding activity and the subsequent derepression of its regulons (Crack et al., 2015; Volbeda et al., 2017). NsrR, as a homodimer, binds to the consensus NsrR-binding site consisting of inverted repeats of two 11 bp motifs (AAxATGCATTT; x, any nucleotide) separated by 1 bp spacing (Partridge et al., 2009; Crack et al., 2015).

The opportunistic human pathogen *Vibrio vulnificus* is a causative agent of foodborne diseases from mild gastroenteritis to primary septicemia (Jones and Oliver, 2009; Baker-Austin and Oliver, 2018). During infection, *V. vulnificus* exploits various transcriptional regulators to sense host-derived signals and modulate the expression of its virulence genes (Miller et al., 1989; Fang et al., 2016). Particularly, a leucine-responsive regulatory protein (Lrp) and a cyclic AMP receptor protein (CRP) are widely conserved and well-characterized global transcriptional regulators in bacteria (Cho et al., 2008; Manneh-Roussel et al., 2018). Lrp controls diverse cellular functions including amino acid metabolism, stress resistance, and virulence (Jeong et al., 2003; Rhee et al., 2008; Lee et al., 2020). The regulatory activity of Lrp on its regulons can be enhanced, reversed, or unaffected by the binding of a small effector molecule leucine (Cho et al., 2008). CRP is a central regulator of carbon and energy metabolism that forms a complex with cyclic AMP (cAMP) (Kim et al., 2011; Lee et al., 2020). In the absence of glucose, the intracellular cAMP level is increased by adenylate cyclase and the resulting cAMP-CRP complex binds DNA to regulate gene expression (Manneh-Roussel et al., 2018). In this way, Lrp and CRP coordinate the expression of genes involved in metabolism and pathogenesis in response to changing environmental conditions such as nutrient availability.

Like many other enteropathogenic bacteria, *V. vulnificus* is inevitably exposed to host-derived nitrosative stress in the course of infection. We recently reported that a multidomain NO dioxygenase HmpA is highly expressed in *V. vulnificus* exposed to NO (Kim et al., 2019). HmpA belongs to the flavohemoglobin family composed of the N-terminal heme-binding globin domain and the C-terminal NAD- and FAD-binding oxidoreductase domain, and detoxifies NO by oxidizing it to a less toxic ${\text{NO}}_{3}^{-}$ under aerobic conditions (Bonamore and Boffi, 2008; Forrester and Foster, 2012; Kim et al., 2019). Because the *in vitro* NO-decomposition activity of *V. vulnificus* is mostly dependent on HmpA, it has a significant role in the survival and pathogenesis of *V. vulnificus* under nitrosative stress in a host (Kim et al., 2019). Nevertheless, definitive regulatory mechanisms and transcriptional regulators, by which *V. vulnificus* senses NO and induces HmpA, have not been yet elucidated in detail. In this study, we newly identified NsrR in *V. vulnificus* as an NO-responsive transcriptional regulator. The transcriptome analysis of the wild-type and isogenic *nsrR*-deletion mutant (Δ*nsrR*) strains revealed that NsrR controls the expression of 47 genes. Notably, *hmpA* was the most highly induced gene by the *nsrR* deletion, indicating that NsrR acts as a strong repressor of *hmpA*. To investigate the exact mechanism by which NsrR regulates *hmpA* expression, the *hmpA* transcript levels were compared in the wild-type and Δ*nsrR* strains under nitrosative stress *in vitro* and *ex vivo*. Furthermore, the combined effect of NsrR, Lrp, and CRP on *hmpA* expression was analyzed at the molecular level. In conclusion, this study suggests that NsrR tightly regulates *hmpA* transcription in response to nitrosative stress together with Lrp and CRP, contributing to the survival and overall success of *V. vulnificus* during host infection.

## Results

### Genome and Transcriptome Analyses Identified NsrR in *V. vulnificus*

**Figure 1:**
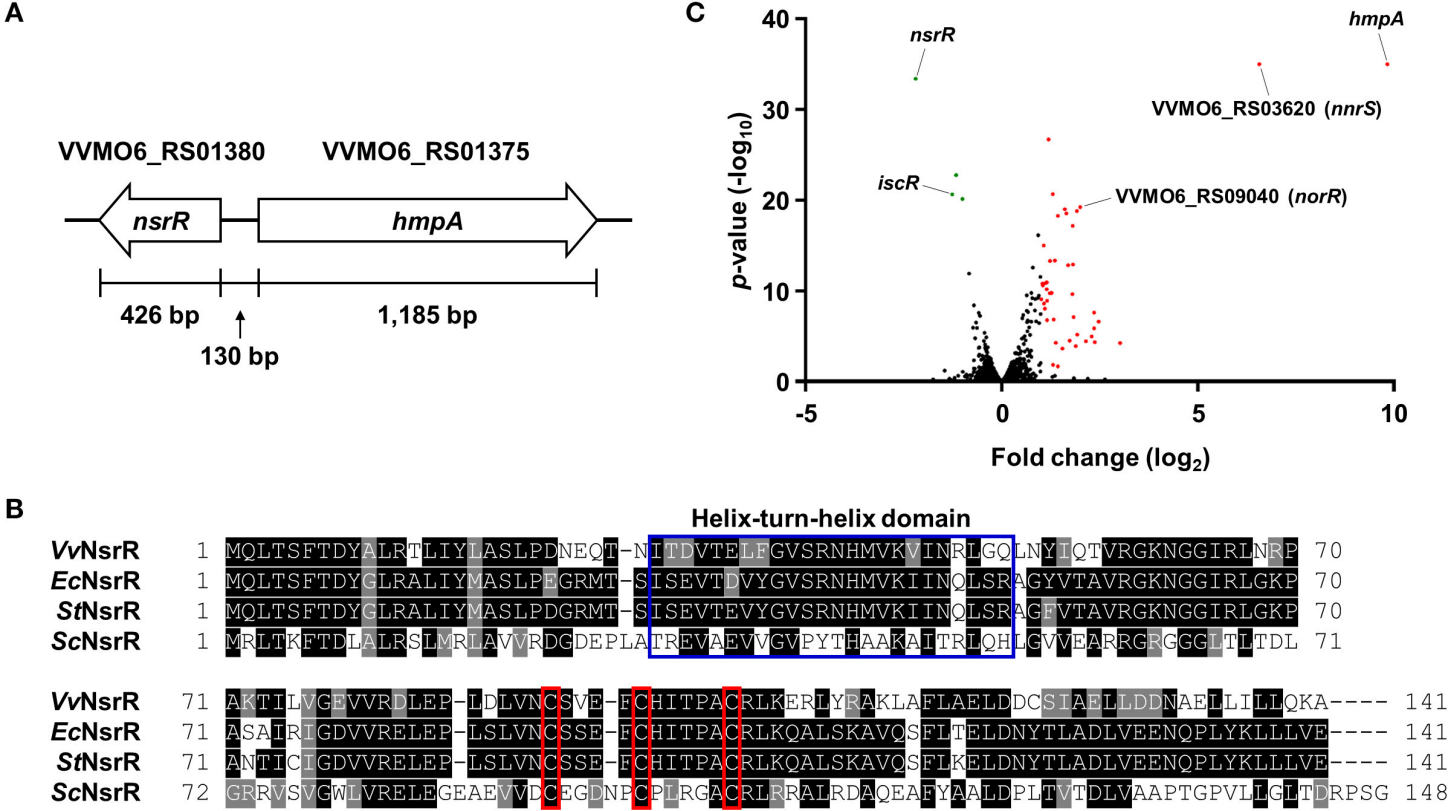
Identification of NsrR and transcriptome analysis of its downstream genes. **(A)** The physical map of *nsrR* and *hmpA* on the *V. vulnificus* MO6-24/O genome. The open arrows represent the coding regions and transcriptional directions of the genes. **(B)** The amino acid sequences of various bacterial NsrRs were retrieved from the NCBI protein database and aligned using the T-Coffee alignment program (Notredame et al., 2000). Identical sequences and conserved sequences are shaded in black and gray, respectively. Dashed lines represent missing sequences. Conserved helix-turn-helix DNA-binding motif and three cysteine residues potentially involved in the Fe-S cluster ligation are indicated by a blue open box and red open boxes, respectively. *Vv*NsrR, *V. vulnificus* NsrR; *Ec*NsrR, *E. coli* NsrR; *St*NsrR, *S*. Typhimurium NsrR; *Sc*NsrR, *S. coelicolor* NsrR. **(C)** The volcano plot depicting the genes differentially expressed by the *nsrR* deletion (fold change ≥ 2 with *p* < 0.05). The red dots and green dots represent the differentially up-regulated and down-regulated genes, respectively.

We previously reported that NO-induced HmpA encoded by VVMO6_RS01375 is crucial for survival under host-derived nitrosative stress and pathogenesis of *V. vulnificus* during infection (Kim et al., 2019). Notably, we further found that the expression of VVMO6_RS01380, which is divergently transcribed from *hmpA* (Figure 1A), is also induced by NO (Kim et al., 2019). VVMO6_RS01380 encodes an Rrf2-family transcriptional regulator showing an amino acid sequence homology to *E. coli* NsrR, *S*. Typhimurium NsrR, and *S. coelicolor* NsrR (61, 62, and 35% identity, respectively) (Figure 1B). Moreover, the protein encoded by VVMO6_RS01380 contains three conserved cysteine residues, C91, C96, and C102, which are known to be essential for the Fe-S cluster ligation of Rrf2-family transcriptional regulators (Figure 1B) (Volbeda et al., 2017). This observation led us to designate the VVMO6_RS01380 gene product as an Fe-S cluster-containing transcriptional regulator NsrR.

For the comprehensive identification of NsrR-regulated genes in *V. vulnificus*, the transcriptomes of the wild-type and Δ*nsrR* strains were compared by RNA-seq. The transcriptome analysis revealed that, in total, 47 genes were differentially expressed by the *nsrR* deletion: 44 genes were up-regulated and 3 genes were down-regulated (Figure 1C, Supplementary Table 1). The overall fold changes of the up-regulated genes were greater than those of the down-regulated genes. This result implies that NsrR serves mainly as a repressor rather than as an activator. Intriguingly, the up-regulated genes included several genes that are predicted to encode proteins involved in the defense against nitrosative stress such as NO dioxygenase HmpA, NO detoxification protein NnrS, ${\text{NO}}_{2}^{-}$ reductase large subunit, ${\text{NO}}_{2}^{-}$ reductase small subunit, cytochrome *c* ${\text{NO}}_{2}^{-}$ reductase subunit *c*_552_ NrfA, and NO reductase transcriptional regulator NorR (Stern et al., 2012; Kim et al., 2019). Among them, *hmpA* was the most highly up-regulated gene in the Δ*nsrR* strain (Figure 1C), suggesting that NsrR is a strong repressor of *hmpA* expression. Meanwhile, the down-regulated genes, *iscR, iscS*, and *iscU*, constitute the *isc* operon (*iscRSUA*-*hscBA*-*fdx*) encoding proteins required for the biogenesis of the Fe-S cluster (Lim and Choi, 2014). Taken together, this result shows that NsrR controls the expression of multiple genes involved in nitrosative stress responses, especially *hmpA*.

### *hmpA* Transcription Is Derepressed by NsrR in Response to NO

**Figure 2:**
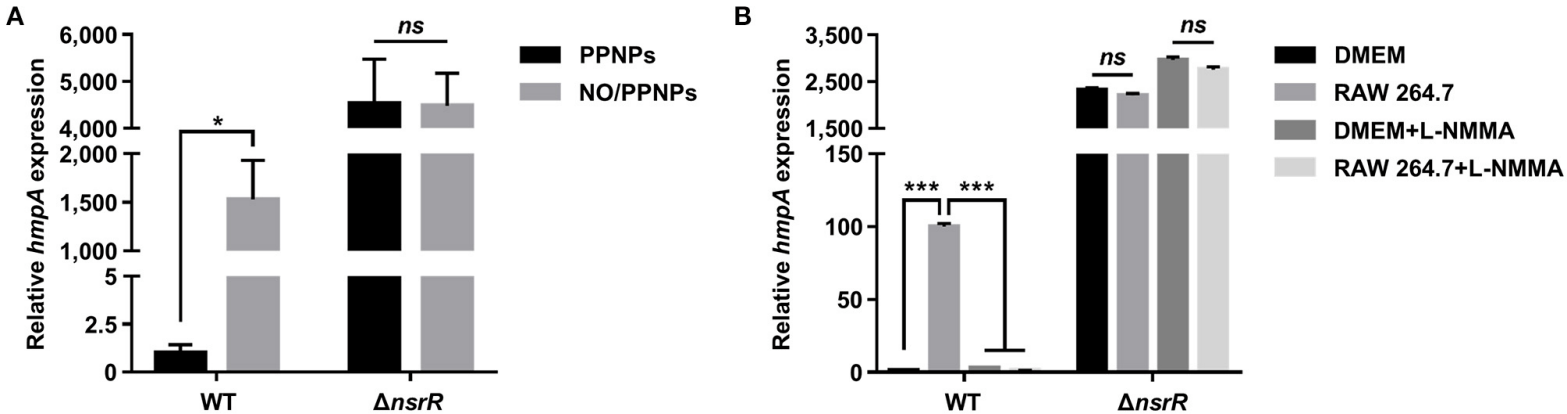
The effect of nitrosative stress and the *nsrR* mutation on *hmpA* transcription *in vitro* and *ex vivo*. The wild-type and Δ*nsrR* strains were grown aerobically to an *A*_600_ of 0.5, and then exposed to 0.15 mg/ml NO/PPNPs *in vitro* **(A)** or NO-producing RAW 264.7 cells *ex vivo* in the presence or absence of L-NMMA **(B)** for 10 min. The *hmpA* transcript levels were determined by qRT-PCR, and the *hmpA* transcript levels in the wild-type strain exposed to PPNPs **(A)** or DMEM without L-NMMA **(B)** were set to 1. Error bars represent the SD. Statistical significance was determined by the Student's *t*-test (*p < 0.05; ***p < 0.0005; *ns*, not significant). WT, wild type; Δ*nsrR, nsrR-*deletion mutant.

To validate the RNA-seq results and examine whether NsrR mediates the induction of *hmpA* in response to NO, the *hmpA* transcript levels in the wild-type and Δ*nsrR* strains were compared under nitrosative stress *in vitro* and *ex vivo*. The *hmpA* transcript level in the wild-type strain was significantly elevated upon exposure to an *in vitro* NO donor, NO/PPNPs (NO-releasing poly(lactic-*co*-glycolic acid)-polyethylenimine nanoparticles) (Figure 2A) (Nurhasni et al., 2015). This result confirms our previous observation that *hmpA* is induced by NO (Kim et al., 2019). Additionally, the *hmpA* transcript level was dramatically increased in the Δ*nsrR* strain compared with that in the wild-type strain even in the absence of NO/PPNPs (Figure 2A), verifying that NsrR negatively regulates *hmpA*. Strikingly, the *hmpA* transcript level in the Δ*nsrR* strain was not affected by the addition of NO/PPNPs (Figure 2A), indicating that NsrR recognizes NO and alleviates the repression of *hmpA* expression *in vitro*.

The role of NsrR in *hmpA* expression was further investigated *ex vivo* using NO-producing murine macrophage RAW 264.7 cells. As shown in Figure 2B, the *hmpA* transcript level in the wild-type strain exposed to NO-producing RAW 264.7 cells was considerably elevated compared with that exposed to Dulbecco's modified Eagle's medium (DMEM; negative control). The extent of the increase in the *hmpA* transcript level upon exposure to the RAW 264.7 cells diminished by the addition of the NO synthase inhibitor L-N^G^-monomethyl arginine citrate (L-NMMA) (Figure 2B). This result suggests that the *hmpA* induction upon exposure to RAW 264.7 cells is attributable to NO produced by the murine macrophages. In contrast, the highly increased *hmpA* transcript level in the Δ*nsrR* strain was not altered by the RAW 264.7 cells and L-NMMA (Figure 2B), confirming that NsrR mediates the derepression of *hmpA* under nitrosative stress derived from host immune cells. The combined results show that NsrR has a critical role to sense NO and to induce the *hmpA* expression both *in vitro* and *ex vivo*.

Then, we examined whether the introduction of recombinant *nsrR* can reduce the increased *hmpA* transcript level in the Δ*nsrR* strain. Introduction of a *nsrR*-expressing plasmid significantly decreased the *hmpA* transcript level, although it was not comparable with that in the wild-type strain (Supplementary Figure 1A). One possible explanation for this lack of complementation is that the recombinant NsrR expressed from the exogenous plasmid is less functional for unknown reasons. On the other hand, ectopic expression of *nsrR* on the chromosome effectively reduced the *hmpA* transcript level comparable with that in the wild-type strain (Supplementary Figure 1B). Similarly, the HmpA protein levels in the Δ*nsrR* strain were highly increased compared with those in the wild-type strain and significantly decreased by complementation (Supplementary Figures 1C,D). Altogether, the results suggest that NsrR is a major transcriptional regulator that recognizes NO and regulates *hmpA* expression mainly at the transcription level.

### Three Conserved Cysteine Residues Are Essential for NsrR to Regulate *hmpA* and *nsrR*

**Figure 3:**
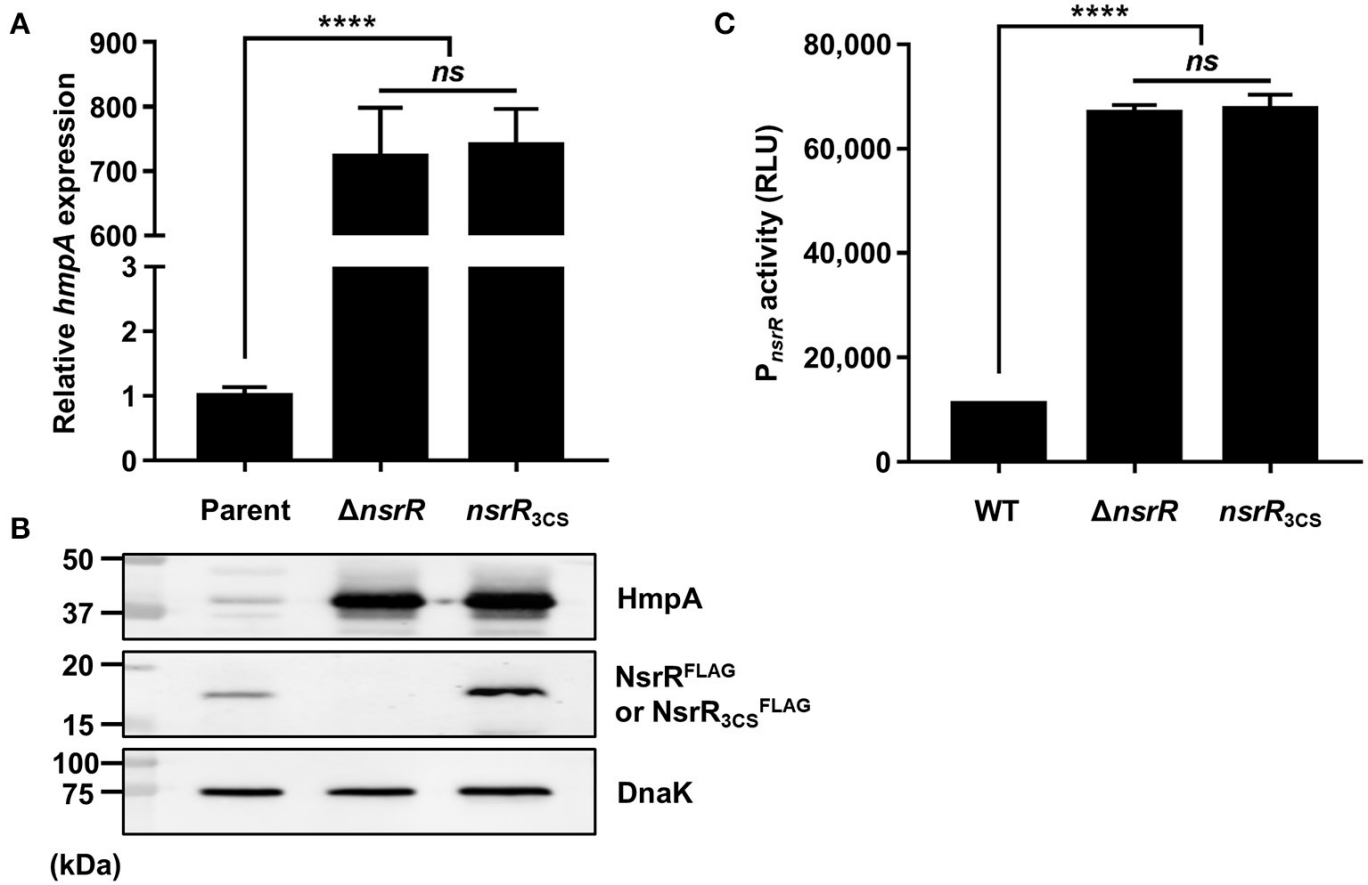
The role of the three cysteine residues in NsrR on *hmpA* and *nsrR* transcription. **(A,B)** Total RNA and proteins were isolated from the parent and mutant strains grown aerobically to an *A*_600_ of 0.5. **(A)** The *hmpA* transcript levels were determined by qRT-PCR, and the *hmpA* transcript level in the parent strain was set to 1. **(B)** The cellular HmpA, NsrR^FLAG^ or ${\text{NsrR}}_{3\text{CS}}^{\text{FLAG}}$, and DnaK (internal control) protein levels were determined by Western blot analysis. Molecular size markers (Bio-Rad) are shown in kDa. Parent, parent strain; Δ*nsrR, nsrR-*deletion mutant; *nsrR*_3CS_, strain expressing apo-locked NsrR^FLAG^. **(C)** A PCR fragment carrying the P_*nsrR*_ was cloned into pBBR-lux to create a reporter plasmid, pGR2025. The wild-type and mutant strains containing pGR2025 were grown aerobically to an *A*_600_ of 0.5, and then used to measure the cellular luminescence. Error bars represent the SD. Statistical significance was determined by the Student's *t*-test (^****^p < 0.00005; *ns*, not significant). RLU, relative luminescence unit; WT, wild type; Δ*nsrR, nsrR*-deletion mutant; *nsrR*_3CS_, strain expressing apo-locked NsrR.

As shown in Figure 1B, *V. vulnificus* NsrR contains three conserved cysteine residues (C91, C96, and C102) that are predicted to act as ligands of the NO-responsive Fe-S cluster (Tucker et al., 2008; Volbeda et al., 2017). To investigate the role of these three cysteine residues, three different strains were constructed: a parent strain GR204 chromosomally encoding 3 × FLAG-tagged NsrR (NsrR^FLAG^), an isogenic *nsrR*-deletion mutant, and an isogenic *nsrR*_3CS_ mutant chromosomally encoding apo-locked NsrR^FLAG^ (${\text{NsrR}}_{3\text{CS}}^{\text{FLAG}}$) (see see Materials and Methods for a detailed description). The *hmpA* transcript and HmpA protein levels in the Δ*nsrR* strain were highly elevated compared with those in the parent strain (Figures 3A,B), indicating that NsrR^FLAG^ in the parent strain is still functional as a repressor of *hmpA*. Notably, the *hmpA* transcript and HmpA protein levels in the *nsrR*_3CS_ strain were comparable with those in the Δ*nsrR* strain (Figures 3A,B). Moreover, a similar effect of the mutation in the three cysteine residues and the *nsrR* deletion on *hmpA* expression was observed in the wild-type background (Supplementary Figure 1E). These results that NsrR_3CS_ cannot repress the *hmpA* transcription reveal that coordination of the Fe-S cluster by the three cysteine residues is essential for the NsrR activity to repress *hmpA*.

**Figure 4:**
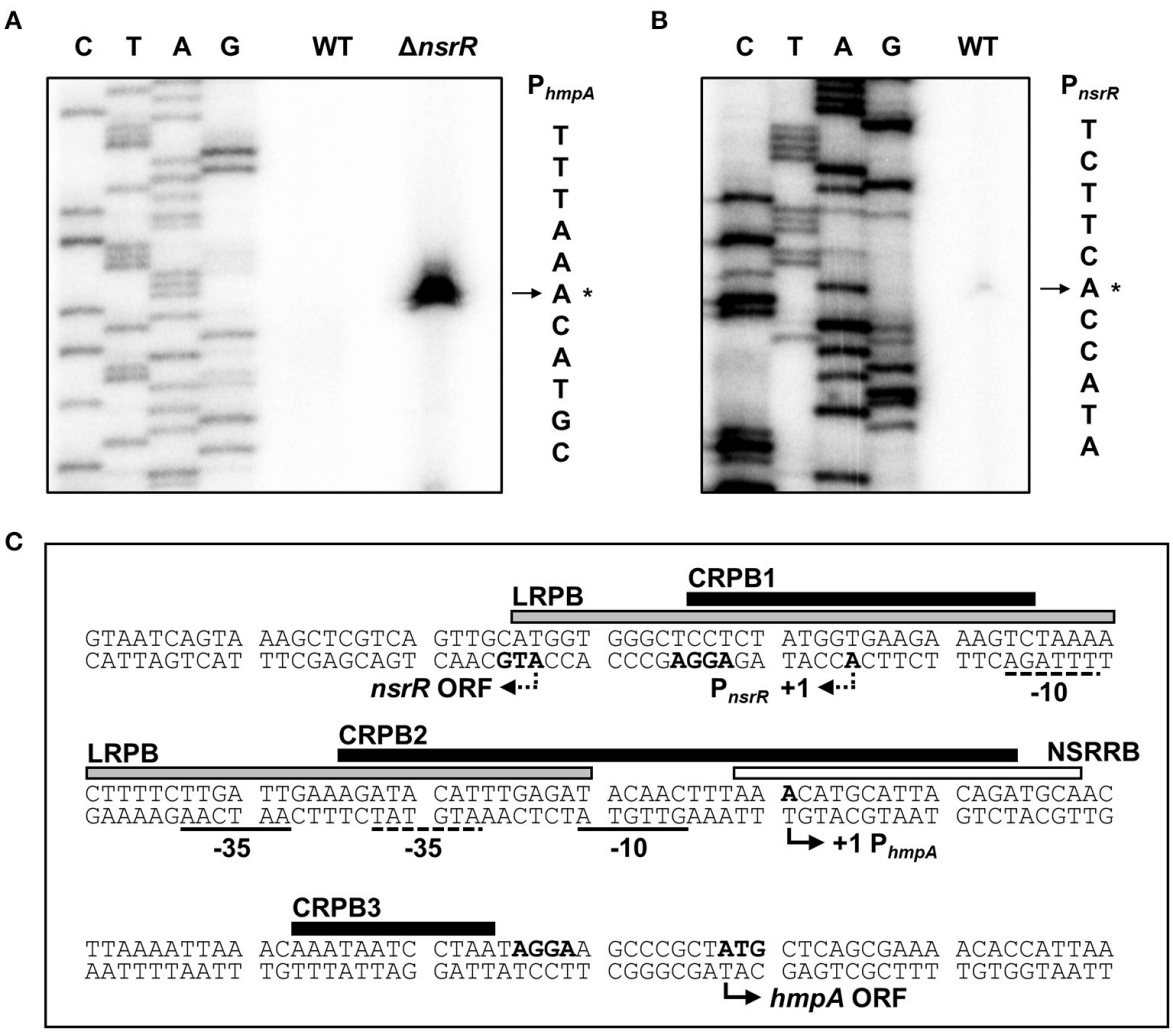
Sequence analysis of the *nsrR*-*hmpA* regulatory region. **(A,B)** The TSSs of *hmpA* **(A)** and *nsrR* **(B)** were determined by the primer extension of RNA isolated from the wild-type and Δ*nsrR* strains grown aerobically to an *A*_600_ of 0.5. Lanes C, T, A, and G represent the nucleotide sequencing ladders. The asterisks indicate the TSSs. WT, wild type; Δ*nsrR, nsrR*-deletion mutant. **(C)** Double-stranded DNA sequence of the *nsrR*-*hmpA* regulatory region is shown. The TSS and putative translation start codon of *nsrR* are indicated by dashed bent arrows, and those of *hmpA* are indicated by solid bent arrows. The putative −10 and −35 regions are underlined with dashed lines for P_*nsrR*_ and solid lines for P_*hmpA*_. The putative ribosome-binding sites (AGGA) are boldface. The binding sequences of NsrR (NSRRB; a white box), Lrp (LRPB; a gray box), and CRP (CRPB1, CRPB2, CRPB3; black boxes) were determined in the later parts of this study.

Furthermore, the ${\text{NsrR}}_{3\text{CS}}^{\text{FLAG}}$ protein level in the *nsrR*_3CS_ strain was significantly elevated compared with the NsrR^FLAG^ protein level in the parent strain (Figure 3B). This observation prompted us to examine the activity of the *nsrR* promoter (P_*nsrR*_, determined in Figures 4B,C) in the wild-type, Δ*nsrR*, and *nsrR*_3CS_ strains using the P_*nsrR*_-*luxCDABE* transcriptional fusion reporter. The P_*nsrR*_ activity in the Δ*nsrR* strain was higher than that in the wild-type strain (Figure 3C), demonstrating that NsrR represses its own transcription. Additionally, the increased P_*nsrR*_ activity in the *nsrR*_3CS_ strain was comparable with that in the Δ*nsrR* strain (Figure 3C). This result suggests that NsrR relieves the repression of its own transcription by the mutation in the three cysteine residues and the consequent loss of the Fe-S cluster. Combined with the previous data (Figure 2), we propose a model in which holo-NsrR containing the Fe-S cluster represses both *hmpA* and *nsrR* transcription, shifts to the clusterless apo-form under nitrosative stress, and then alleviates the repression of *hmpA* and *nsrR* transcription.

### P*_*hmpA*_* and P*_*nsrR*_* Overlap Divergently With Each Other

To map the *hmpA* promoter, the transcription start site (TSS) of *hmpA* was determined by primer extension analysis. A single reverse transcript was produced from the primer extension of RNA isolated from the Δ*nsrR* strain grown to an *A*_600_ of 0.5 (Figure 4A). This reverse transcript observed in the Δ*nsrR* strain was not detected in the wild-type strain (Figure 4A), confirming that the *hmpA* transcription is strongly repressed by NsrR. The 5′-end of *hmpA* was located 57-bp upstream of the translation start codon of *hmpA*. Next, the TSS of *nsrR* was determined in a similar way. A single reverse transcript was produced from the primer extension of RNA isolated from the wild-type strain grown to an *A*_600_ of 0.5 (Figure 4B). The 5′-end of *nsrR* was located 18-bp upstream of the translation start codon of *nsrR*. The putative promoters constituting the TSSs were named P_*hmpA*_ and P_*nsrR*_ to represent the *hmpA* promoter and the *nsrR* promoter, respectively. The sequences for putative −10 and −35 regions of each promoter were assigned based on the similarity to the consensus sequences of *E. coli* σ^70^ promoters (Figure 4C). Strikingly, these results show that P_*hmpA*_ and P_*nsrR*_ overlap with each other. This overlapping promoter region was termed the *nsrR*-*hmpA* regulatory region for our further research.

### NsrR Directly Binds to the *nsrR*-*hmpA* Regulatory Region to Repress *hmpA* and Its Own Expression

**Figure 5:**
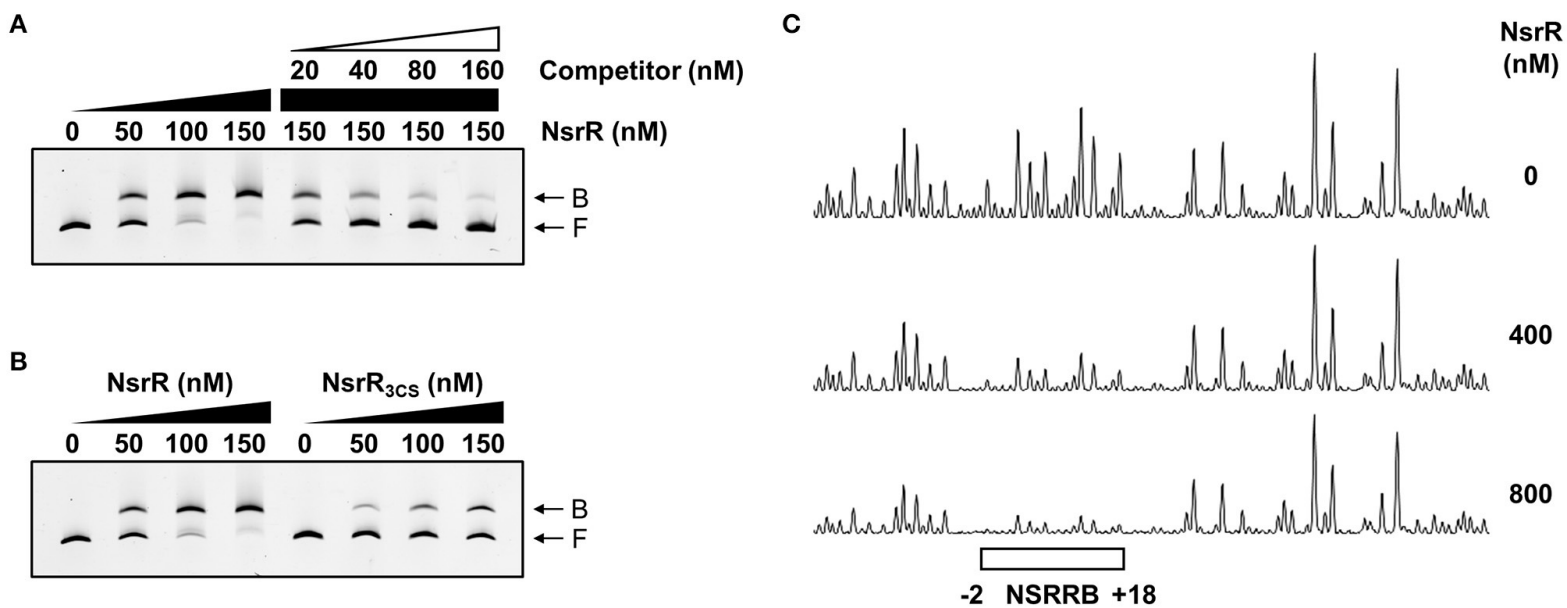
Specific binding of NsrR to the *nsrR*-*hmpA* regulatory region. **(A,B)** A 393-bp DNA fragment of the *nsrR*-*hmpA* regulatory region (10 nM) was labeled with 6-FAM, and then incubated with increasing amounts of NsrR **(A,B)** or NsrR_3CS_ **(B)** as indicated. For the competition analysis, various amounts of the unlabeled DNA fragment were added as a self-competitor. B, bound DNA; F, free DNA. **(C)** The same DNA probe (40 nM) was incubated with increasing amounts of NsrR as indicated, and then digested with DNase I. The region protected by NsrR is indicated by a white box (NSRRB). Nucleotide numbers shown are relative to the TSS of *hmpA*.

To investigate whether NsrR directly binds to the *nsrR*-*hmpA* regulatory region, electrophoretic mobility shift assays (EMSAs) were performed. The addition of NsrR to 6-carboxyfluorescein (6-FAM)-labeled DNA probe encompassing the *nsrR*-*hmpA* regulatory region resulted in a single retarded band in an NsrR concentration-dependent manner (Figure 5A). The same unlabeled DNA fragment competed for NsrR binding in a dose-dependent manner (Figure 5A), confirming the specific binding of NsrR. Then, the binding of NsrR_3CS_ to the *nsrR*-*hmpA* regulatory region was compared with that of NsrR. The amount of the retarded band of the DNA-NsrR_3CS_ complex was reduced compared with that of the DNA-NsrR complex (Figure 5B). This result implies that the DNA-binding affinity of NsrR_3CS_ is considerably lower than that of NsrR, which leads to the derepression of *hmpA* and *nsrR* under nitrosative stress.

To determine the precise location of NsrR-binding site(s) in the *nsrR*-*hmpA* regulatory region, DNase I protection assays were performed using the same DNA probe. When NsrR was added to the DNA probe, NsrR protected a single region extending from −2 to +18 (NSRRB, centered at +8.5 from the TSS of *hmpA*) from DNase I digestion (Figures 4C, 5C). The sequence of NSRRB showed about 87% similarity to the 11-1-11 bp consensus NsrR-binding sequence in *E. coli* (Bodenmiller and Spiro, 2006; Partridge et al., 2009). Combined with the EMSA data (Figure 5A), these results indicate that NsrR concurrently represses *hmpA* and its own transcription by directly binding to the single specific sequence in the *nsrR*-*hmpA* regulatory region.

### Lrp Represses *hmpA* in an NsrR-Dependent Manner by Directly Binding to the *nsrR*-*hmpA* Regulatory Region

**Figure 6:**
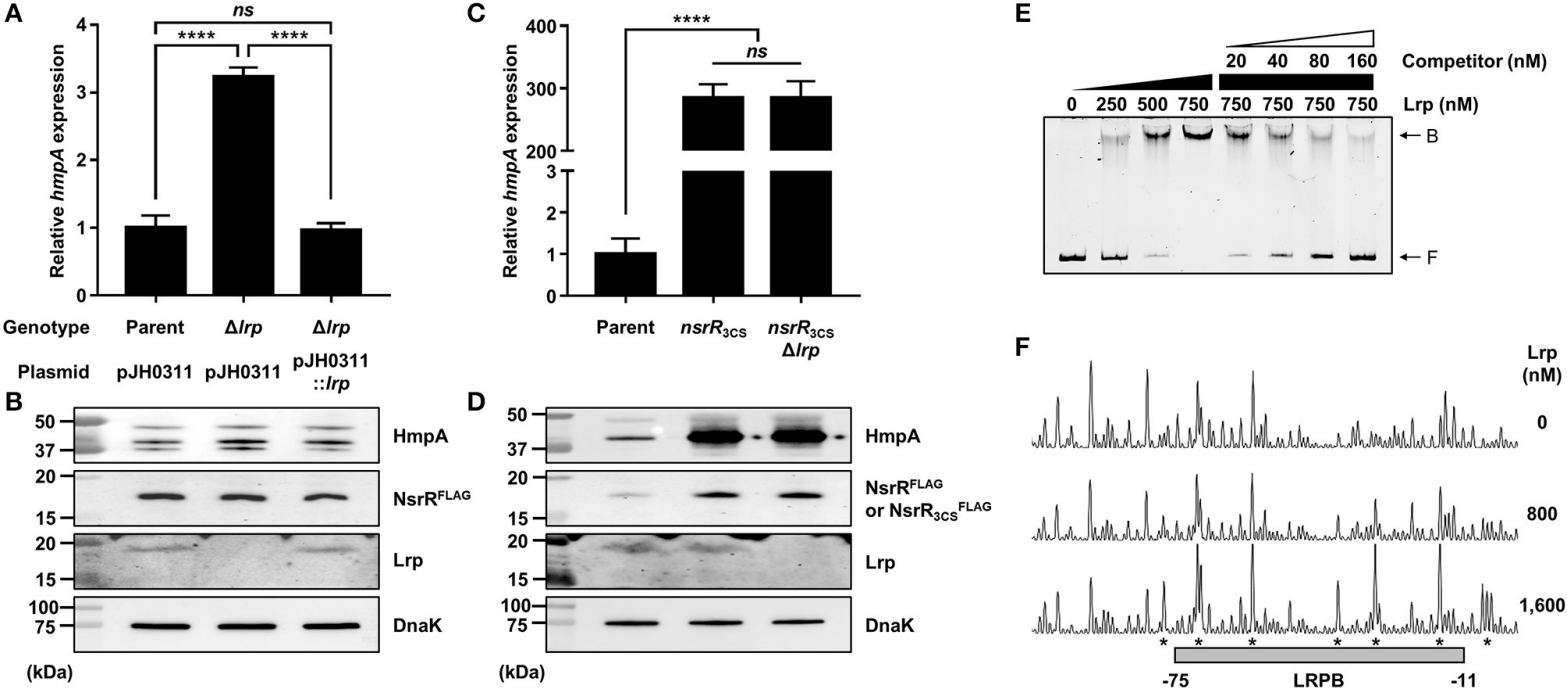
The effect of the *lrp* mutation on *hmpA* transcription and the specific binding of Lrp to the *nsrR*-*hmpA* regulatory region. **(A–D)** Total RNA and proteins were isolated from the parent and mutant strains grown aerobically to an *A*_600_ of 0.5. **(A,C)** The *hmpA* transcript levels were determined by qRT-PCR, and the *hmpA* transcript levels in the parent strain were set to 1. Error bars represent the SD. Statistical significance was determined by the Student's *t*-test (^****^p < 0.00005; *ns*, not significant). **(B,D)** The cellular HmpA, NsrR^FLAG^ or ${\text{NsrR}}_{3\text{CS}}^{\text{FLAG}}$, Lrp, and DnaK (internal control) protein levels were determined by Western blot analysis. Molecular size markers (Bio-Rad) are shown in kDa. Parent, parent strain; Δ*lrp, lrp*-deletion mutant; pJH0311, broad-host-range vector; pJH0311::*lrp*, pJH0311 carrying the *lrp* gene (pZW1818); *nsrR*_3CS_, strain expressing apo-locked NsrR^FLAG^; *nsrR*_3CS_Δ*lrp, lrp*-deletion mutant expressing apo-locked NsrR^FLAG^. **(E)** A 393-bp DNA fragment of the *nsrR*-*hmpA* regulatory region (10 nM) was labeled with 6-FAM, and then incubated with increasing amounts of Lrp as indicated. For the competition analysis, various amounts of the unlabeled DNA fragment were added as a self-competitor. B, bound DNA; F, free DNA. **(F)** The same DNA probe (40 nM) was incubated with increasing amounts of Lrp as indicated, and then digested with DNase I. The region protected by Lrp is indicated by a gray box (LRPB). The nucleotides showing enhanced cleavage are indicated by asterisks. Nucleotide numbers shown are relative to the TSS of *hmpA*.

To determine other factors involved in the *hmpA* regulation, we further explored various known transcriptional regulators in *V. vulnificus*. Among them, the role of Lrp in the *hmpA* regulation was evaluated. The *hmpA* transcript and HmpA protein levels in the *lrp*-deletion mutant (Δ*lrp*) were significantly increased compared with those in the parent strain and restored by complementation (Figures 6A,B). The *hmpA* transcript level in the wild-type strain was not altered by exogenous leucine (Supplementary Figure 2A), suggesting that Lrp negatively affects the *hmpA* transcription in a leucine-independent manner. To investigate the regulatory relationship between NsrR and Lrp, the *lrp*-deleted *nsrR*_3CS_ mutant (*nsrR*_3CS_Δ*lrp*), in which both NsrR and Lrp are not functional, was constructed from the parent strain. Interestingly, the *hmpA* transcript and HmpA protein levels in the *nsrR*_3CS_Δ*lrp* strain were comparable with those in the *nsrR*_3CS_ strain (Figures 6C,D). The observation that Lrp was not able to affect *hmpA* transcription in the absence of functional NsrR indicates that the negative effect of Lrp on *hmpA* is mediated by NsrR. This result led us to examine whether Lrp positively regulates the cellular level of NsrR to repress *hmpA*. However, both the P_*nsrR*_ activity and NsrR^FLAG^ protein level were not affected by the *lrp* deletion (Supplementary Figure 2B, Figure 6B).

Next, EMSAs were performed to investigate whether Lrp directly binds to the *nsrR*-*hmpA* regulatory region. The addition of Lrp to the DNA probe resulted in a single retarded band in an Lrp concentration-dependent manner (Figure 6E). The same unlabeled DNA fragment showed competition for Lrp binding in a dose-dependent manner (Figure 6E), demonstrating the specific binding of Lrp. DNase I protection assays revealed that Lrp largely protected a single region extending from −75 to −11 (LRPB, centered at −43 from the TSS of *hmpA*) from DNase I digestion (Figures 4C, 6F). Combined with the EMSA data (Figure 6E), these results indicate that Lrp binds directly and specifically to the *nsrR*-*hmpA* regulatory region. Notably, within the region protected by Lrp, a periodic pattern of reduced cleavage followed by short regions of enhanced cleavage was observed (Figure 6F). This pattern known as phased hypersensitivity implies DNA bending by a multimeric Lrp (Pul et al., 2007), suggesting that the Lrp multimer induces a conformation change of the *nsrR*-*hmpA* regulatory region. Moreover, EMSA with both NsrR and Lrp showed that NsrR and Lrp simultaneously bind to the *nsrR-hmpA* regulatory region, rather than displace each other (Supplementary Figure 2C). Altogether, the combined results propose that direct binding of Lrp to the *nsrR*-*hmpA* regulatory region does not alter the *nsrR* transcription but represses *hmpA* transcription presumably through the modification of the DNA conformation enhancing the *hmpA* repression by NsrR.

### CRP Activates *hmpA*, but Represses *nsrR* by Directly Binding to the *nsrR*-*hmpA* Regulatory Region

**Figure 7:**
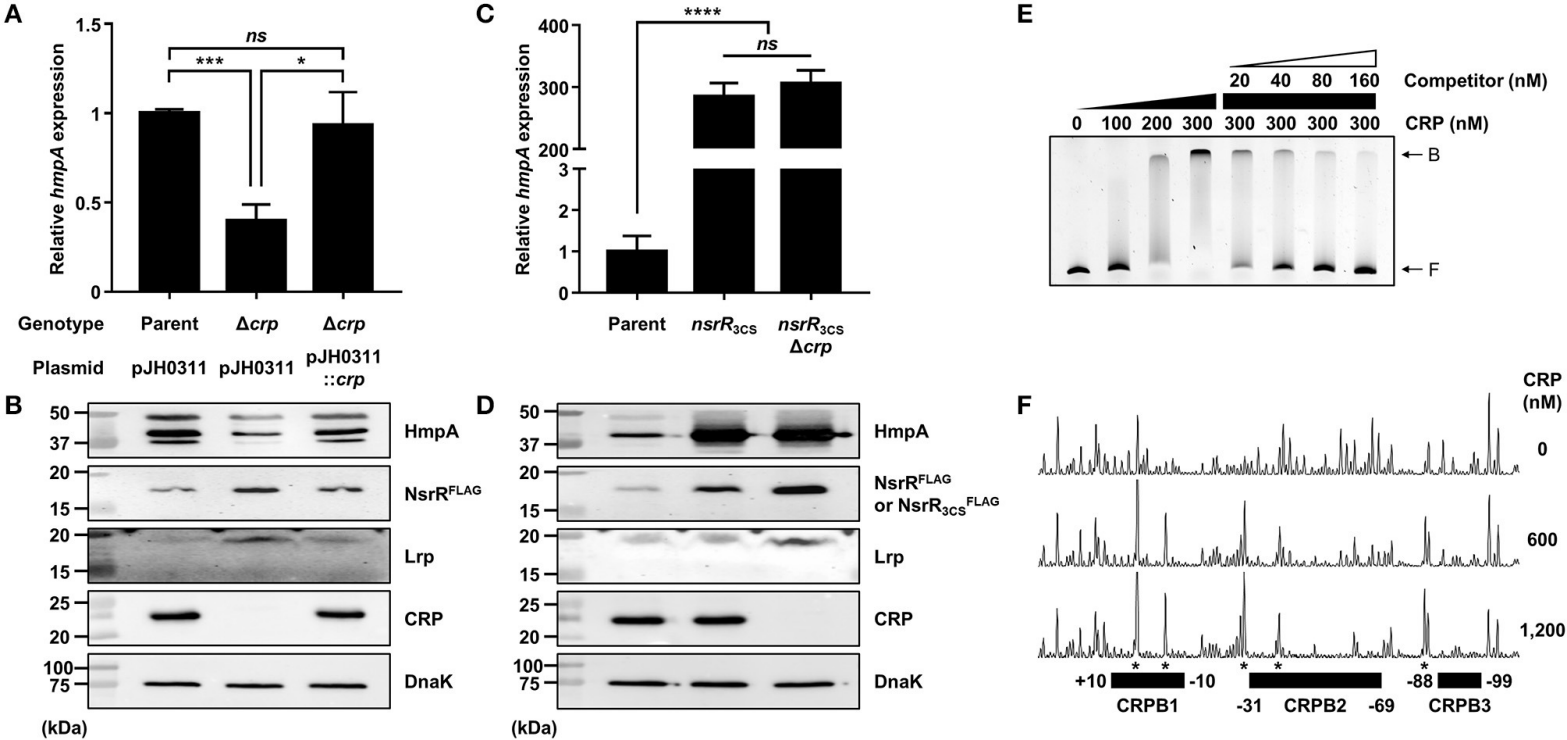
The effect of the *crp* mutation on *hmpA* and *nsrR* transcription, and the specific binding of CRP to the *nsrR*-*hmpA* regulatory region. **(A–D)** Total RNA and proteins were isolated from the parent and mutant strains grown aerobically to an *A*_600_ of 0.5. **(A,C)** The *hmpA* transcript levels were determined by qRT-PCR, and the *hmpA* transcript levels in the parent strain were set to 1. Error bars represent the SD. Statistical significance was determined by the Student's *t-*test (*p < 0.05; ***p < 0.0005; ****p < 0.00005; *ns*, not significant). **(B,D)** The cellular HmpA, NsrR^FLAG^ or ${\text{NsrR}}_{3\text{CS}}^{\text{FLAG}}$, Lrp, CRP, and DnaK (internal control) protein levels were determined by Western blot analysis. Molecular size markers (Bio-Rad) are shown in kDa. Parent, parent strain; Δ*crp, crp*-deletion mutant; pJH0311, broad-host-range vector; pJH0311::*crp*, pJH0311 carrying the *crp* gene (pKK1502); *nsrR*_3CS_, strain expressing apo-locked NsrR^FLAG^; *nsrR*_3CS_Δ*crp, crp*-deletion mutant expressing apo-locked NsrR^FLAG^. **(E)** A 393-bp DNA fragment of the *nsrR*-*hmpA* regulatory region (10 nM) was labeled with 6-FAM, and then incubated with increasing amounts of CRP as indicated. For the competition analysis, various amounts of the unlabeled DNA fragment were added as a self-competitor. B, bound DNA; F, free DNA. **(F)** The same DNA probe (40 nM) was incubated with increasing amounts of CRP as indicated, and then digested with DNase I. The regions protected by CRP are indicated by block boxes (CRPB1, CRPB2, and CRPB3). The nucleotides showing enhanced cleavage are indicated by asterisks. Nucleotide numbers shown are relative to the TSS of *nsrR*.

The role of CRP in the *hmpA* regulation was also explored. The *hmpA* transcript and HmpA protein levels in the *crp*-deletion mutant (Δ*crp*) were considerably decreased compared with those in the parent strain and restored by complementation (Figures 7A,B). In addition, the *hmpA* transcript level in the wild-type strain was decreased by exogenous glucose while that in the Δ*crp* strain was not affected (Supplementary Figure 3A). These results indicate that CRP has a positive effect on the *hmpA* transcription which is relieved in the presence of exogenous glucose. Then, we compared the *hmpA* transcript and HmpA protein levels in the parent strain, the *nsrR*_3CS_ strain, and the *crp*-deleted *nsrR*_3CS_ mutant (*nsrR*_3CS_Δ*crp*). Similar to Lrp, the *hmpA* transcript and HmpA protein levels in the *nsrR*_3CS_Δ*crp* strain were comparable with those in the *nsrR*_3CS_ strain (Figures 7C,D), suggesting that the positive effect of CRP on the *hmpA* transcription is also mediated by NsrR. Thus, we further examined whether the effect of CRP on *hmpA* expression results from the increased cellular level of NsrR. Notably, the P_*nsrR*_ activity and NsrR^FLAG^ protein level were significantly increased by the *crp* deletion (Supplementary Figure 3B, Figure 7B), showing that CRP acts as a repressor of *nsrR* transcription. Moreover, the Lrp protein level in the Δ*crp* strain was elevated compared with that in the parent strain as we observed previously (Figure 7B) (Lee et al., 2020). Accordingly, we hypothesized that CRP indirectly activates *hmpA* through the repression of both *nsrR* and *lrp* in a sequential manner.

To investigate whether CRP directly binds to the *nsrR*-*hmpA* regulatory region, EMSAs were performed. As shown in Figure 7E, the addition of CRP to the DNA probe resulted in a single retarded band in a CRP concentration-dependent manner. The same unlabeled DNA fragment competed for CRP binding in a dose-dependent manner (Figure 7E), confirming the specific binding of CRP. DNase I protection assays determined three regions protected by CRP extending from −10 to +10 (CRPB1, centered at −0.5 from the TSS of *nsrR*), −69 to −31 (CRPB2, centered at −50 from the TSS of *nsrR*), and −99 to −88 (CRPB3, centered at −93.5 from the TSS of *nsrR*) from DNase I digestion (Figures 4C, 7F). Combined with the EMSA data showing a single retarded band by CRP (Figure 7E), this result implies that CRP binds to CRPB1, CRPB2, and CRPB3 with similar DNA-binding affinities. Taken together, the combined results propose that CRP directly and specifically binds to the *nsrR*-*hmpA* regulatory region to repress *nsrR* as well as *lrp*, and consequently induces the *hmpA* transcription in a sequential cascade. In conclusion, the results in this study suggest that NsrR tightly regulates the *hmpA* transcription in response to NO, which could be elaborated by Lrp and CRP.

## Discussion

In this study, we newly identified and characterized an NO-responsive transcriptional regulator NsrR in *V. vulnificus* (Figure 1). The transcriptome analysis discovered that 44 genes are negatively regulated and 3 genes are positively regulated by NsrR (Supplementary Table 1). Notably, our previous transcriptome analysis of the wild-type strain revealed that 42 of the 44 genes repressed by NsrR are significantly induced upon exposure to NO (Supplementary Figure 4A) (Kim et al., 2019). Among the 42 genes, we further identified that the induction of *nnrS*, in addition to *hmpA*, is mediated by NsrR in response to NO (Figure 2, Supplementary Figure 4B). Accordingly, it is possible to propose that NsrR also regulates the expression of various genes other than *hmpA* and *nnrS* by sensing nitrosative stress. Meanwhile, although *iscR, iscS*, and *iscU* were positively regulated by NsrR (Supplementary Table 1), NsrR did not directly bind to the promoter region of the *isc* operon under the conditions tested (Supplementary Figure 5), indicating that NsrR controls the *isc* operon indirectly. Considering that functional NsrR requires the intact Fe-S cluster, up-regulation of the *isc* operon would be advantageous for NsrR to control its regulons effectively.

Besides *nsrR*, we found that the expression of *norR*, encoding another putative NO-responsive transcriptional regulator NorR, is induced by NO (Kim et al., 2019). In *V. cholerae*, NorR activates *hmpA* and *nnrS* by sensing NO, contributing to NO detoxification and the sustained colonization of host intestines (Stern et al., 2012). However, NorR did not affect the *hmpA* transcription in *V. vulnificus* under our experimental conditions (Supplementary Figure 6). Meanwhile, NorR in *E. coli* is known to activate the *norVW* genes encoding a flavorubredoxin to detoxify NO (D'Autreaux et al., 2005), but we could not find *norVW* homologs in the *V. vulnificus* genome. Although NorR-regulated genes and their role require further studies, NsrR appears to be the major transcriptional regulator for *V. vulnificus* to respond against nitrosative stress so far.

**Figure 8:**
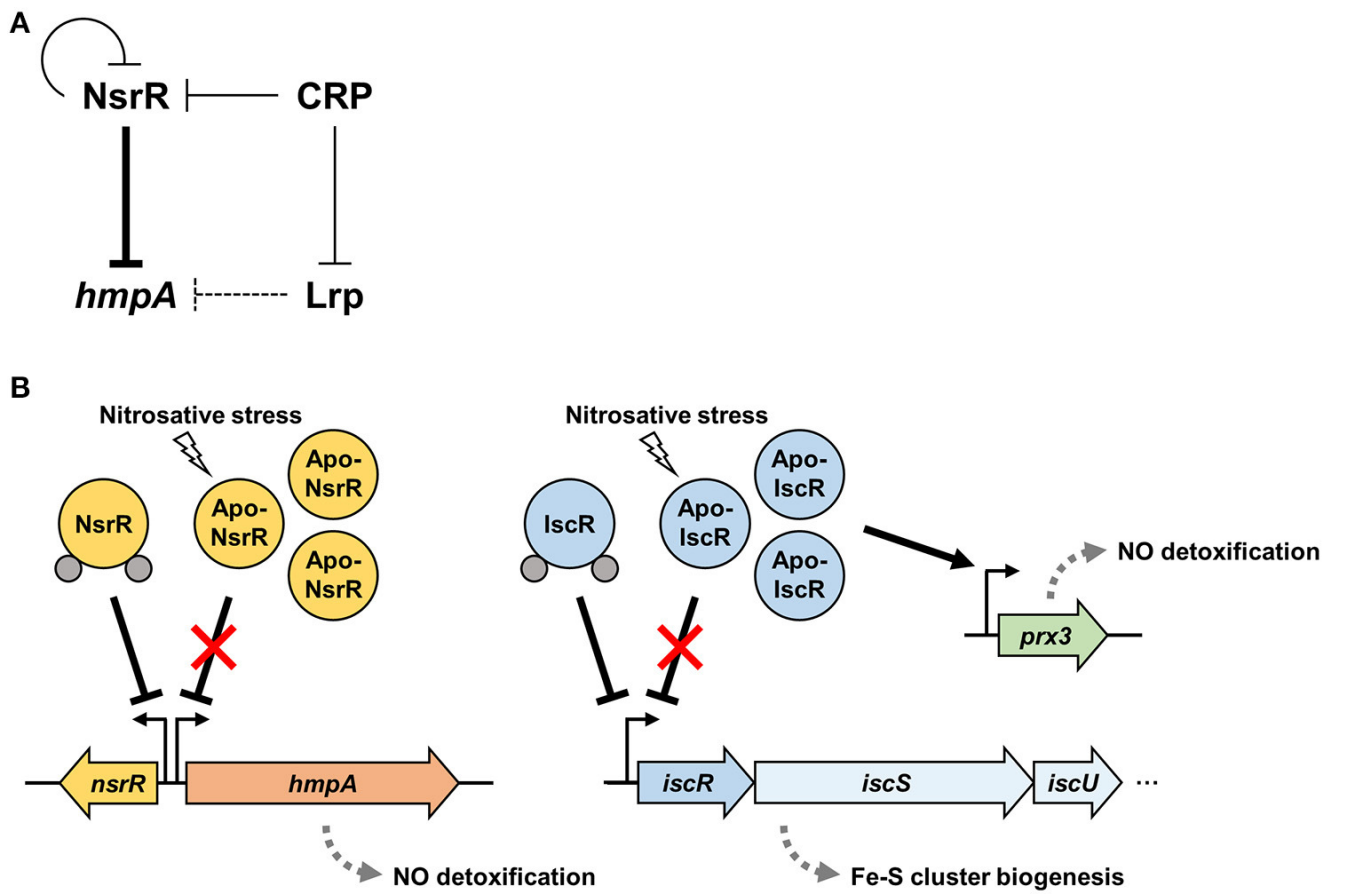
A regulatory network controlling *hmpA* transcription and nitrosative stress defense systems in *V. vulnificus*. **(A)** A regulatory network comprising transcriptional regulators NsrR, Lrp, and CRP controls the *hmpA* transcription. NsrR directly represses *hmpA* and *nsrR* itself. Lrp indirectly represses *hmpA* presumably by enhancing the repression activity of NsrR through DNA structure remodeling. CRP indirectly activates *hmpA* possibly through the repression of *nsrR* and *lrp* in a sequential cascade. **(B)** Apo-NsrR alleviates the repression of *hmpA* and *nsrR* upon exposure to nitrosative stress. On the other hand, apo-IscR relieves the repression of the *isc* operon, and the resulting increased apo-IscR directly activates *prx3* encoding 1-cysteine peroxiredoxin. The induced NO-decomposition proteins, HmpA and Prx3, would facilitate the survival of *V. vulnificus* under host-derived nitrosative stress. The gray dots represent the Fe-S cluster.

Figure 8A depicts the regulatory network comprising NsrR, Lrp, and CRP for the *hmpA* transcription proposed by this study. NsrR relieves the direct repression of *hmpA* losing its Fe-S cluster and DNA-binding affinity under nitrosative stress (Figures 3, 5). The strong repression of *hmpA* by NsrR could allow *V. vulnificus* to prevent unnecessary waste of cellular components such as heme, NAD, and FAD as cofactors of HmpA (Kim et al., 2019). On the other hand, it could facilitate the rapid and strong induction of *hmpA* when the repression by NsrR is abolished, which may ensure an effective response against nitrosative stress (Alon, 2007). Thus, it is tempting to suppose that NsrR has evolved to regulate *hmpA* transcription by a derepression mechanism rather than simple activation.

Furthermore, Lrp and CRP elaborate the *hmpA* regulation by functional NsrR. Lrp directly binds to the *nsrR*-*hmpA* regulatory region but is not able to repress *hmpA* in the absence of functional NsrR (Figure 6). As one of the bacterial nucleoid-associated proteins, Lrp can modulate gene expression by remodeling the DNA structure (Dillon and Dorman, 2010). Thus, one possible explanation for the NsrR-dependent *hmpA* repression by Lrp is that the formation of a multicomponent complex containing Lrp multimers and the resulting conformation change of DNA enhance the ability of holo-NsrR to repress *hmpA*. Meanwhile, this study further demonstrated that CRP acts as a repressor of *nsrR* by directly binding to the *nsrR*-*hmpA* regulatory region (Figure 7, Supplementary Figure 3B). In addition, we confirmed our previous report that CRP directly represses *lrp* by binding to its promoter (Figure 7B) (Lee et al., 2020). These results led us to propose that CRP activates *hmpA* by the repression of *nsrR* and *lrp* as a sequential cascade.

Particularly, we showed that CRP upregulates the *hmpA* transcription in response to low levels of glucose (Supplementary Figure 3A). During conditions of intestinal inflammation, NO produced by host cells is rapidly decomposed to less toxic ${\text{NO}}_{3}^{-}$ by diverse detoxifying enzymes of enteric pathogens including HmpA. The accumulated ${\text{NO}}_{3}^{-}$ in the intestinal lumen can be utilized as an electron acceptor for anaerobic respiration of pathogens in hypoxic environments (Vazquez-Torres and Baumler, 2016; Bueno et al., 2018). In the ${\text{NO}}_{3}^{-}$/${\text{NO}}_{2}^{-}$ respiration, ${\text{NO}}_{3}^{-}$ is converted to ${\text{NO}}_{2}^{-}$ that is harmful to bacteria. Thus, ${\text{NO}}_{2}^{-}$ is subsequently reduced to ammonia (NH_3_) by an ${\text{NO}}_{2}^{-}$ reductase, which can generate NO as a by-product (Spiro, 2007; Tiso and Schechter, 2015). Intriguingly, it has been reported that CRP activates the ${\text{NO}}_{3}^{-}$/${\text{NO}}_{2}^{-}$ respiration under nutrient-poor or low-oxygen conditions in *E. coli* and *Shewanella oneidensis* (Stewart et al., 2009; Dong et al., 2012). Accordingly, we could assume that CRP induces *hmpA* as well as activates ${\text{NO}}_{3}^{-}$/${\text{NO}}_{2}^{-}$ respiration under low-glucose conditions to scavenge the low levels of endogenous NO during ${\text{NO}}_{3}^{-}$/${\text{NO}}_{2}^{-}$ respiration. Since the utilization of host-derived ${\text{NO}}_{3}^{-}$ enhances the growth and fitness of pathogens (Vazquez-Torres and Baumler, 2016), NsrR and CRP might coordinate nitrosative stress defense systems and energy production in *V. vulnificus* for survival during infection. Altogether, the collaborative regulation by NsrR along with Lrp and CRP enables the tight and precise tuning of *hmpA* transcription by integrating various signals including nitrosative stress and nutrient availability, thereby contributing to the fitness and pathogenesis of *V. vulnificus* within the host.

Our current understanding of the nitrosative stress defense systems in *V. vulnificus* is summarized in Figure 8B. In addition to NsrR, we previously demonstrated that *V. vulnificus* IscR, another Rrf2-family [2Fe-2S] containing transcriptional regulator, also turns to an apo-form lacking the Fe-S cluster under nitrosative stress (Lim and Choi, 2014; Choi et al., 2020). Apo-IscR dissociates from the promoter of the *isc* operon to express the *isc* operon and to facilitate the biogenesis of the Fe-S cluster (Lim et al., 2014b). In addition, the resulting increased apo-IscR further activates the expression of *prx3* encoding 1-cysteine peroxiredoxin with an NO-decomposition activity by directly binding to the *prx3* promoter region (P_*prx*3_) (Lim et al., 2014a; Ahn et al., 2018). The regulatory characteristic of IscR on P_*prx*3_ is distinguishable from that of NsrR on P_*hmpA*_ in which IscR can bind to P_*prx*3_ in the apo-form, and the increased apo-IscR protein level results in *prx3* activation. Taken together, these assorted nitrosative stress defense systems would provide *V. vulnificus* with the benefit of having inclusive modulation of various NO-detoxifying gene expression and the consequent survival under host-derived nitrosative stress during infection.

## Materials and Methods

### Strains, Plasmids, and Culture Conditions

The strains and plasmids used in this study are listed in Supplementary Table 2. Unless otherwise noted, the *V. vulnificus* strains were grown aerobically in Luria-Bertani (LB) medium supplemented with 2% (w/v) NaCl (LBS) at 30°C, and their growth was monitored spectrophotometrically at 600 nm (*A*_600_). When required, 3 μg/ml chloramphenicol was added to the media. To visualize the cellular NsrR protein levels, *V. vulnificus* GR204, which carries 3 × FLAG-coding sequence fused to the 3′-end of *nsrR* ORF on the chromosome, was constructed as a parent strain (Supplementary Table 2). The parent strain and its isogenic mutants were used to quantify the cellular NsrR protein levels. The murine macrophage RAW 264.7 cells were grown in DMEM containing 10% fetal bovine serum (VWR, Radnor, PA) and the antibiotics [100 units/ml penicillin G and 100 μg/ml streptomycin (Gibco-BRL, Gaithersburg, MD)] in air supplemented with 5% CO_2_ at 37°C. To induce NO production, the RAW 264.7 cells were suspended in fresh DMEM containing 500 ng/ml *E. coli* O111:B4 lipopolysaccharide (Sigma, St. Louis, MO) and 1 mM L-arginine (Sigma) (Walker et al., 1997; Choi et al., 2020).

### Generation and Complementation of the Mutants

For construction of the isogenic deletion mutants, target genes were inactivated *in vitro* by deletion of each ORF using the PCR-mediated linker-scanning mutation method as described previously (Jang et al., 2016; Choi et al., 2020). Briefly, the deleted ORF fragment was amplified by PCR with appropriate primer pairs (Supplementary Table 3), and the resulting fragment was ligated into SphI-SpeI-digested pDM4 (Milton et al., 1996). *E. coli* S17-1 λ*pir* (Simon et al., 1983) containing pDM4 with the desired insert was used as a conjugal donor to an appropriate *V. vulnificus* strain to generate the deletion mutant (Supplementary Table 2). The conjugation and isolation of the transconjugants were conducted using a method described previously (Choi et al., 2020). The *lrp*-deletion mutant ZW181 and the *crp*-deletion mutant DI0201 were constructed previously (Choi et al., 2002; Lee et al., 2020).

For construction of the parent strain GR204 encoding NsrR^FLAG^ on the chromosome, the 3 × FLAG-coding sequence was fused to the 3'-end of *nsrR* ORF by PCR using the primer pairs NSRR01-F and NSRR01F-R, or NSRR02F-F and NSRR02-R (Supplementary Table 3). The amplified fragment was cloned into pDM4, resulting in pGR2008 (Supplementary Table 2). *E. coli* S17-1 λ*pir* containing pGR2008 was used as a conjugal donor to the Δ*nsrR* strain as described above to generate GR204 (Supplementary Table 2).

The three cysteine residues in NsrR (C91, C96, and C102) were replaced with serine to examine their regulatory function with the minimal structural change of NsrR. For construction of the *nsrR*_3CS_ strain DY192, three cysteine residues were substituted with serine *in vitro* by using the QuikChange® site-directed mutagenesis kit (Agilent Technologies, Loveland, CO) (Bang et al., 2012; Lim et al., 2014a). The complementary mutagenic primers listed in Supplementary Table 3 were used to create pDY1907 carrying the *nsrR*_3CS_ gene on pDM4 (Supplementary Table 2). *E. coli* S17-1 λ*pir* containing pDY1907 was used as a conjugal donor to the Δ*nsrR* strain as described above to generate DY192, and the *nsrR*_3CS_ mutation in DY192 was confirmed by DNA sequencing. For construction of the 3 × FLAG-tagged *nsrR*_3CS_ strain GR217 (Supplementary Table 2), a similar method was adopted except using pGR2016 carrying 3 × FLAG-coding sequence fused to the 3′-end of *nsrR*_3CS_ ORF on pDM4 instead of pDY1907.

To complement the *nsrR* mutation with a plasmid-based system, the *nsrR* gene was amplified by PCR using the primer pair NSRRC-F and -R (Supplementary Table 3). The amplified fragment was cloned into the broad-host-range vector pJH0311 (Goo et al., 2006) to create pDY1702 (Supplementary Table 2). To complement the *lrp* and *crp* mutation, pZW1818 and pKK1502 carrying the *lrp* and *crp* gene on pJH0311, respectively, were used in this study (Supplementary Table 2) (Jang et al., 2017; Lee et al., 2020). The plasmids were transferred into appropriate mutants by conjugation as described above.

To complement the *nsrR* mutation by ectopic expression of *nsrR* on the chromosome, the *nsrR* regulatory region and its ORF was integrated into a cryptic *lacZ* gene by PCR using specific primer pairs listed in Supplementary Table 3 (Hall, 1999; Chodur et al., 2017). The amplified fragment was cloned into pDM4, resulting in pGR2007 (Supplementary Table 2). *E. coli* S17-1 λ*pir* containing pGR2007 was used as a conjugal donor to the Δ*nsrR* strain as described above to generate GR203 (Supplementary Table 2).

### RNA-seq and Transcriptome Analysis

To analyze the effect of the *nsrR* deletion on the *V. vulnificus* transcriptome, total RNA was isolated from biological duplicates of the wild-type and Δ*nsrR* strains, grown aerobically to an *A*_600_ of 0.5 in M9 minimal media supplemented with 0.4% (w/v) glucose (M9G) and then exposed to PPNPs for 10 min (Nurhasni et al., 2015; Kim et al., 2019). The RNA was further purified by removing DNA using TURBO DNase (Ambion, Austin, TX), and mRNA was selectively enriched by depleting rRNA using a Ribo-Zero rRNA removal kit (Epicenter, Madison, WI) according to the manufacturer's instructions. Strand-specific cDNA libraries were constructed and sequenced using HiSeq 2500 (Illumina, San Diego, CA) as described previously (Lee et al., 2019). The raw sequencing reads were mapped to the *V. vulnificus* MO6-24/O reference genome (GenBank^TM^ accession numbers: CP002469 and CP002470, www.ncbi.nlm.nih.gov), and the expression level of each gene was calculated as a reads per kilobase of transcript per million mapped sequence reads (RPKM) value using EDGE-pro v1.3.1 (Estimated Degree of Gene Expression in PROkaryots) (Magoc et al., 2013). The RPKM values were normalized and analyzed statistically using DeSeq2 v1.26.0 to identify the differentially expressed genes (fold change ≥ 2 with *p* < 0.05) (Love et al., 2014). A heat map was generated by the Matplotlib python package using the RPKM-fold change for each gene (Hunter, 2007).

### qRT-PCR and Primer Extension Analysis

Relative transcript levels in the total RNA isolated from the *V. vulnificus* strains grown under various environmental conditions were determined by quantitative RT-PCR (qRT-PCR). In detail, *V. vulnificus* was grown to an *A*_600_ of 0.5 in M9G and then exposed to either 0.15 mg/ml PPNPs (negative control) or NO/PPNPs for 10 min (Nurhasni et al., 2015; Kim et al., 2019). Additionally, *V. vulnificus* grown to an *A*_600_ of 0.5 in LBS was exposed to DMEM (negative control) or RAW 264.7 cells at a multiplicity of infection 10 for 10 min in the presence or absence of 500 μM L-NMMA (Sigma), which is a known NO synthase inhibitor (Nathan and Hibbs, 1991; Choi et al., 2020). When necessary, *V. vulnificus* was grown to an *A*_600_ of 0.5 in LBS with various amounts of L-leucine (Sigma) or 1% glucose (Sigma). Total RNA from the *V. vulnificus* cells was isolated and quantified using a RNeasy® Mini Kit (Qiagen, Valencia, CA) and a NanoDrop One^c^ Microvolume UV-Vis Spectrophotometer (Thermo Fisher Scientific, Waltham, MA), respectively. cDNA was synthesized from 500 ng of the total RNA with the iScript^TM^ cDNA synthesis kit (Bio-Rad, Hercules, CA). Real-time PCR amplification of the cDNA was performed with the Chromo 4 real-time PCR detection system (Bio-Rad) and specific primer pairs (Supplementary Table 3) as described previously (Jang et al., 2017). Relative expression levels were calculated with the 16S rRNA expression level as an internal reference for normalization (Jang et al., 2017).

For primer extension analysis, primers HMPAUP-R and NSRRUP-R (Supplementary Table 3) complementary to the coding region of *hmpA* and *nsrR*, respectively, were end-labeled with [γ-32P]-ATP and added to the RNA. The primers were extended with SuperScript II reverse transcriptase (Invitrogen, Carlsbad, CA). The cDNA products were purified and resolved on a sequencing gel alongside sequencing ladders generated from pDY1706 and pDY1707 (Supplementary Table 2) with the same primers, respectively. The plasmid pDY1706 was constructed by cloning the 219-bp *hmpA* upstream region extending from −120 to +99, amplified by PCR using a primer pair HMPAUP-F and -R (Supplementary Table 3), into pGEM-T Easy (Promega, Madison, WI). Similarly, pDY1707 carrying the 198-bp *nsrR* upstream region extending from −113 to +85 on pGEM-T Easy was constructed using a primer pair NSRRUP-F and -R (Supplementary Table 3). The primer extension product was visualized with the Typhoon FLA 7000 phosphorimager (GE healthcare, Menlo Park, CA).

### Protein Purification and Western Blot Analysis

To overexpress NsrR and NsrR_3CS_, each ORF of *nsrR* and *nsrR*_3CS_ was amplified by PCR using specific primer pairs (Supplementary Table 3). The amplified fragments were cloned into pET-28a(+) (Novagen, Madison, WI) to create pEJ1902 and pEJ1903, respectively (Supplementary Table 2). The resulting His_6_-tagged NsrR and NsrR_3CS_ were expressed in *E. coli* BL21 (DE3) and purified by affinity chromatography according to the manufacturer's instructions (Qiagen). The buffers used for NsrR and NsrR_3CS_ are as follows: 20 mM Tris-Cl (pH 8.0), 500 mM NaCl, and 5 mM β-mercaptoethanol; additional 10% glycerol for a lysis buffer; additional 20 mM imidazole for a wash buffer; additional 250 mM imidazole for an elution buffer; additional 50% glycerol for a dialysis buffer. To overexpress Lrp and CRP, pZW1903 carrying the *lrp* gene on pET-28a(+) and pHK0201 carrying the *crp* gene on pRSET A (Invitrogen) were used in this study (Supplementary Table 2) (Choi et al., 2002; Lee et al., 2020). The His_6_-tagged Lrp and CRP were purified as described previously (Lee et al., 2020).

For Western blot analysis, *V. vulnificus* cells were lysed using B-PER^TM^ Bacterial Protein Extraction Reagent with Enzymes (Thermo Fisher Scientific), and residual cell debris was removed by centrifugation to obtain clear cell lysates. The protein levels of HmpA, Lrp, CRP, and DnaK in the clear cell lysates were determined as described previously (Kim et al., 2019; Lee et al., 2020). Similarly, cellular NsrR^FLAG^ protein was detected using Monoclonal ANTI-FLAG® M2 antibody produced in mouse (Sigma).

### Construction of P*_*nsrR*_*-*luxCDABE* Transcriptional Fusion

A 393-bp *nsrR-hmpA* regulatory region (−262 to +131 from the TSS of *nsrR*) was amplified with the primer PnsrR-F carrying a SacI restriction site and PnsrR-R carrying a SpeI restriction site (Supplementary Table 3). The resulting DNA fragment was cloned into the SacI-SpeI-digested pBBR-lux carrying the promoterless *luxCDABE* genes to create pGR2025 (Supplementary Table 2) (Lenz et al., 2004). pGR2025 was transferred into the *V. vulnificus* strains by conjugation as described above. The cellular luminescence and growth (*A*_600_) of each strain grown to an *A*_600_ of 0.5 in LBS were measured using a microplate reader (Infinite^TM^ microplate reader, Tecan, Männedorf, Switzerland), and RLUs were calculated by dividing the luminescence with the *A*_600_ (Lee et al., 2019).

### EMSA and DNase I Protection Assay

For the EMSAs, a 393-bp *nsrR*-*hmpA* regulatory region (−186 to +207 from the TSS of *hmpA*, equivalent to −262 to +131 from the TSS of *nsrR*) was amplified by PCR using 6-FAM-labeled PnsrRhmpA-F and -R as primers (Supplementary Table 3). Similarly, a 321-bp *isc* operon regulatory region [−194 to +127 from the TSS of *isc* operon (Lim et al., 2014b)] was amplified by PCR using 6-FAM-labeled Pisc-F and -R as primers (Supplementary Table 3). The 6-FAM-labeled DNA probe (10 nM) was then incubated with purified NsrR or CRP for 30 min at 30°C in a 20-μl reaction mixture containing 1 × NsrR binding buffer (10 mM Tris-Cl (pH 8.0), 10 mM KCl, 1 mM DTT, and 100 μg BSA; additional 1 mM cAMP only for CRP) and 0.1 μg of poly(dI-dC) (Sigma) as a non-specific competitor. Similarly, the DNA probe was incubated with purified Lrp or both NsrR and Lrp for 30 min at 30°C in a 20-μl reaction mixture containing 1 × Lrp binding buffer (50 mM Tris-Cl (pH 8.0), 20 mM KCl, 1 mM DTT, and 100 μg BSA, and 10% glycerol) and 0.1 μg of poly(dI-dC) (Sigma) as a non-specific competitor. For the competition analysis, various concentrations of unlabeled DNA fragment were added as a self-competitor to the reaction mixture before incubation. Electrophoretic analysis of the DNA-protein complexes was performed as described previously (Lee et al., 2020).

The same 393-bp *nsrR*-*hmpA* regulatory region was amplified by PCR using unlabeled PnsrRhmpA-F and 6-FAM-labeled PnsrRhmpA-R as primers for the DNase I protection assays (Supplementary Table 3). The binding of NsrR, Lrp, and CRP to the DNA probe (40 nM) was performed as described above, and DNase I digestion of the DNA-protein complexes followed the procedures described previously (Jang et al., 2017). The digested DNA products were precipitated with ethanol and eluted in sterilized H_2_O, and then analyzed using an ABI 3730xl DNA analyzer (Applied Biosystems, Foster City, CA) with Peak Scanner^TM^ Software v1.0 (Applied Biosystems) (Hwang et al., 2019).

### Data Analysis

Average and standard deviation (SD) values were calculated from at least three independent experiments. Statistical analysis was performed by the Student's *t*-test using GraphPad Prism 7.0 (GraphPad Software, San Diego, CA).

## Data Availability Statement

The raw data of the RNA-seq analysis can be found in the NCBI BioProject database–PRJNA704465; https://www.ncbi.nlm.nih.gov/bioproject/PRJNA704465.

## Author Contributions

GC, DK, and SC designed the research. GC and DK performed the experiments. GC and SC wrote the manuscript. All authors analyzed, interpreted the data, reviewed the results, and approved the final version of the manuscript.

## Conflict of Interest

The authors declare that the research was conducted in the absence of any commercial or financial relationships that could be construed as a potential conflict of interest.
