# Supplementary material for: A Nitric Oxide-Responsive Transcriptional Regulator NsrR Cooperates With Lrp and CRP to Tightly Control the hmpA Gene in Vibrio vulnificus
Source: Front Microbiol. 2021 May 21;12:681196. doi: 10.3389/fmicb.2021.681196 (PMC8175989; doi:10.3389/fmicb.2021.681196)
Supplement: Supplementary file 1 [file Table_1.pdf]

Supplementary Table 1. The genes differentially expressed by the *nsrR* deletion revealed from the transcriptome analysis in this study.

Locus tag[a]	Fold change (log_2)	Gene product
**Up-regulated (44 genes)**		
VVMO6_RS01375	9.83	Flavohemoprotein / Hemoglobin-like protein / Flavohemoglobin / Nitric oxide dioxygenase
VVMO6_RS03620	6.57	NnrS protein involved in response to NO
VVMO6_RS23435	3.02	Hypothetical protein
VVMO6_RS17305	2.47	Alcohol dehydrogenase
VVMO6_RS10920	2.37	Hypothetical protein
VVMO6_RS19095	2.36	Nitrite reductase [NAD(P)H] large subunit
VVMO6_RS03320	2.36	Putative protease
VVMO6_RS05915	2.29	Putative transporter
VVMO6_RS05050	2.15	Hypothetical protein
VVMO6_RS09040	2.00	Functional role page for anaerobic nitric oxide reductase transcription regulator NorR
VVMO6_RS19825	1.93	Tripeptide aminopeptidase
VVMO6_RS19090	1.92	Nitrite reductase [NAD(P)H] small subunit
VVMO6_RS01180	1.89	Succinate dehydrogenase flavoprotein subunit
VVMO6_RS10915	1.84	Ferrous iron transport protein A
VVMO6_RS08640	1.82	Peptidase, M20A family
VVMO6_RS09395	1.81	Dethiobiotin synthetase
VVMO6_RS00655	1.80	Hypothetical protein
VVMO6_RS10200	1.74	Alcohol dehydrogenase / acetaldehyde dehydrogenase
VVMO6_RS03315	1.69	Putative protease
VVMO6_RS16865	1.65	Hypothetical protein
VVMO6_RS19085	1.61	Nitrite transporter from formate/nitrite family
VVMO6_RS09810	1.55	Cytochrome c552 precursor
VVMO6_RS16860	1.44	Ferric siderophore transport system biopolymer transport protein ExbB
VVMO6_RS03330	1.44	Putative lipid carrier protein
VVMO6_RS08610	1.38	Hypothetical protein
VVMO6_RS19080	1.36	Uroporphyrinogen-III methyltransferase
VVMO6_RS05920	1.32	Sensor histidine kinase
VVMO6_RS09400	1.31	8-amino-7-oxononanoate synthase
VVMO6_RS16870	1.30	Radical SAM family protein HutW, coproporphyrinogen III oxidase-like protein, oxygen-independent, associated with heme uptake
VVMO6_RS16855	1.27	Biopolymer transport protein ExbD1

VVMO6_RS16850	1.23	Periplasmic hemin-binding protein
VVMO6_RS17945	1.23	Ferric vibriobactin, enterobactin transport system, permease protein VctG
VVMO6_RS01385	1.20	Cytosine deaminase
VVMO6_RS09865	1.16	Glycine cleavage system regulatory protein
VVMO6_RS05810	1.15	Hypothetical protein
VVMO6_RS17940	1.15	Ferric vibriobactin, enterobactin transport system, permease protein VctD
VVMO6_RS16845	1.15	Hemin ABC transporter permease protein
VVMO6_RS20630	1.12	ABC transporter, ATP-binding protein
VVMO6_RS08325	1.10	Ferredoxin-type protein NapG (periplasmic nitrate reductase)
VVMO6_RS20625	1.08	Hypothetical protein
VVMO6_RS09515	1.07	Iron-regulated protein A precursor
VVMO6_RS18940	1.05	Ribonucleotide reductase of class III (anaerobic) large subunit
VVMO6_RS09510	1.04	Probable thiol oxidoreductase with 2 cytochrome c heme-binding sites
VVMO6_RS18655	1.01	Uncharacterized paraquat-inducible protein B
**Down-regulated (3 genes)**		
VVMO6_RS12225	-1.26	Iron-sulfur cluster regulator IscR
VVMO6_RS12220	-1.16	Cysteine desulfurase IscS subfamily
VVMO6_RS12215	-1.00	Iron-sulfur cluster assembly scaffold protein IscU

[a] Locus tags are based on the *V. vulnificus* MO6-24/O genome (GenBank™ accession numbers: CP002469 and CP002470, www.ncbi.nlm.nih.gov).
