# Supplementary material for: A Nitric Oxide-Responsive Transcriptional Regulator NsrR Cooperates With Lrp and CRP to Tightly Control the hmpA Gene in Vibrio vulnificus
Source: Front Microbiol. 2021 May 21;12:681196. doi: 10.3389/fmicb.2021.681196 (PMC8175989; doi:10.3389/fmicb.2021.681196)
Supplement: Supplementary file 2 [file Table_2.pdf]

Supplementary Table 2. Bacterial strains and plasmids used in this study.

Strain or plasmid	Relevant characteristics[a]	Reference or source
**Bacterial strains**		
V. vulnificus		
MO6-24/O	Wild-type strain; clinical isolate; virulent	Laboratory collection
DY172	MO6-24/O with Δ*nsrR*	This study
GR203	DY172 with ectopic integration of *nsrR*	This study
DY192	MO6-24/O with $nsrR_{3CS}$ encoding the apo-locked NsrR	This study
ZW181	MO6-24/O with Δ*lrp*	(Lee et al., 2020)
DI0201	MO6-24/O with Δ*crp*	(Choi et al., 2002)
DY161	MO6-24/O with Δ*norR*	This study
GR204	Parent strain; MO6-24/O with *nsrR*-3×FLAG encoding the 3×FLAG-tagged NsrR	This study
GR207	GR204 with Δ*nsrR*	This study
GR217	GR204 with $nsrR_{3CS}$ encoding the apo-locked NsrR-3×FLAG	This study
GR222	GR204 with Δ*lrp*	This study
GR228	GR204 with $nsrR_{3CS}$Δ*lrp*	This study
GR208	GR204 with Δ*crp*	This study
GR227	GR204 with $nsrR_{3CS}$Δ*crp*	This study
E. coli		
S17-1 λ*pir*	λ-*pir* lysogen; *thi pro hsdR* $hsdM^+$ *recA* RP4-2 Tc::Mu-Km::Tn7; Tp^r Sm^r; host for π-requiring plasmids; conjugal donor	(Simon et al., 1983)
BL21 (DE3)	F^- *ompT* $hsdS_B$ (r_B^- m_B^-) *gal dcm* (DE3)	Laboratory collection
**Plasmids**		
pDM4	Suicide vector; R6K γ ori *sacB*; *oriT* of RP4; Cm^r	(Milton et al., 1996)
pDY1621	pDM4 with Δ*nsrR*; Cm^r	This study
pGR2007	pDM4 with *nsrR*; Cm^r	This study
pDY1907	pDM4 with $nsrR_{3CS}$; Cm^r	This study
pZW1817	pDM4 with Δ*lrp*; Cm^r	(Lee et al., 2020)
pBS0907	pDM4 with Δ*crp*; Cm^r	(Kim et al., 2011)
pDY1615	pDM4 with Δ*norR*; Cm^r	This study
pGR2008	pDM4 with *nsrR*-3×FLAG; Cm^r	This study
pGR2013	pDM4 with Δ*nsrR*-3×FLAG; Cm^r	This study
pGR2016	pDM4 with $nsrR_{3CS}$-3×FLAG; Cm^r	This study
pJH0311	Broad-host-range vector; 0.3-kb MCS of pUC19 cloned into pCOS5; ColEI *oriV*; *oriT* of RK2; Ap^r, Cm^r	(Goo et al., 2006)
pDY1702	pJH0311 with *nsrR*; Ap^r, Cm^r	This study
pZW1818	pJH0311 with *lrp*; Ap^r, Cm^r	(Lee et al., 2020)

pKK1502	pJH0311 with *crp*; Ap^r, Cm^r	(Jang et al., 2017)
pGEM-T Easy	PCR product cloning vector; Ap^r	Promega
pDY1706	pGEM-T Easy with 219-bp fragment of *hmpA* upstream region; Ap^r	This study
pDY1707	pGEM-T Easy with 198-bp fragment of *nsrR* upstream region; Ap^r	This study
pET-28a(+)	His$_6$-tag fusion protein expression vector; Km^r	Novagen
pEJ1902	pET-28a(+) with *nsrR*; Km^r	This study
pEJ1903	pET-28a(+) with *nsrR*$_{3CS}$; Km^r	This study
pZW1903	pET-28a(+) with *lrp*; Km^r	(Lee et al., 2020)
pHK0201	pRSET A with *crp*; Ap^r	(Choi et al., 2002)
pBBR-lux	Broad-host-range vector; promoterless *luxCDABE* cloned into pBBR1MCS; pBBR1 *oriV*; Cm^r	(Lenz et al., 2004)
pGR2025	pBBR-lux with 393-bp fragment of *nsrR* regulatory region	This study

[a] Tp^r, trimethoprim-resistant; Sm^r, streptomycin-resistant; Cm^r, chloramphenicol-resistant; Ap^r, ampicillin-resistant; Km^r, kanamycin-resistant; MCS, multiple cloning site.

REFERENCES

Choi, H. K., Park, N. Y., Kim, D. I., Chung, H. J., Ryu, S., and Choi, S. H. (2002). Promoter analysis and regulatory characteristics of *vvhBA* encoding cytolytic hemolysin of *Vibrio vulnificus*. *J. Biol. Chem.* 277**,** 47292-47299. doi: 10.1074/jbc.M206893200

Goo, S. Y., Lee, H. J., Kim, W. H., Han, K. L., Park, D. K., Lee, H. J., et al. (2006). Identification of OmpU of *Vibrio vulnificus* as a fibronectin-binding protein and its role in bacterial pathogenesis. *Infect. Immun.* 74**,** 5586-5594. doi: 10.1128/IAI.00171-06

Jang, K. K., Lee, Z. W., Kim, B., Jung, Y. H., Han, H. J., Kim, M. H., et al. (2017). Identification and characterization of *Vibrio vulnificus plpA* encoding a phospholipase A_2 essential for pathogenesis. *J. Biol. Chem.* 292**,** 17129-17143. doi: 10.1074/jbc.M117.791657

Kim, B. S., Hwang, J., Kim, M. H., and Choi, S. H. (2011). Cooperative regulation of the *Vibrio vulnificus nan* gene cluster by NanR protein, cAMP receptor protein, and *N*-acetylmannosamine 6-phosphate. *J. Biol. Chem.* 286**,** 40889-40899. doi: 10.1074/jbc.M111.300988

Lee, Z. W., Hwang, S. H., Choi, G., Jang, K. K., Lee, T. H., Chung, K. M., et al. (2020). A MARTX toxin *rtxA* gene is controlled by host environmental signals through a CRP-coordinated regulatory network in *Vibrio vulnificus*. *mBio* 11:e00723-20. doi: 10.1128/mBio.00723-20

Lenz, D. H., Mok, K. C., Lilley, B. N., Kulkarni, R. V., Wingreen, N. S., and Bassler, B. L. (2004). The small RNA chaperone Hfq and multiple small RNAs control quorum sensing in *Vibrio harveyi* and *Vibrio cholerae*. *Cell* 118**,** 69-82. doi: 10.1016/j.cell.2004.06.009

Milton, D. L., O'toole, R., Horstedt, P., and Wolf-Watz, H. (1996). Flagellin A is essential for the virulence of *Vibrio anguillarum*. *J. Bacteriol.* 178**,** 1310-1319. doi: 10.1128/JB.178.5.1310-1319.1996

Simon, R., Priefer, U., and Puhler, A. (1983). A broad host range mobilization system for *in vivo* genetic-engineering - transposon mutagenesis in Gram-negative bacteria. *Bio-Technology* 1**,** 784-791. doi: 10.1038/nbt1183-784
