# Supplementary material for: A Nitric Oxide-Responsive Transcriptional Regulator NsrR Cooperates With Lrp and CRP to Tightly Control the hmpA Gene in Vibrio vulnificus
Source: Front Microbiol. 2021 May 21;12:681196. doi: 10.3389/fmicb.2021.681196 (PMC8175989; doi:10.3389/fmicb.2021.681196)
Supplement: Supplementary file 3 [file Table_3.pdf]

Supplementary Table 3. Oligonucleotides used in this study.

Primer	Oligonucleotide sequence, 5' →3'[a, b]	Use
**For mutant construction**		
NSRR01-F	GAGCTCAGGTTACCCGCATGCTATTGTCCTGGTTTGAAGCTA	
NSRR01-R	CGTTGTCATCATCAGTAAAGCTCGTCAGTTGCAT	
NSRR02-F	CTTTACTGATGATGACAACGCAGAGCTCCTGATC	
NSRR02-R	CGACCCTCGAGTACGCGTCACTAGTGCCATGCCACCACTCAAAGTGATC	Deletion of *nsrR* ORF and 3×FLAG fusion to *nsrR* ORF
NSRR01F-R	GTCGATGTCATGATCTTTATAATCACCGTCATGGTCTTTGTAGTCGGCTTTTTGCAGCAAGATC	
NSRR02F-F	GATTATAAAGATCATGACATCGACTACAAAGACGATGACGACAAGTAACTGGCGTTGCCAGTATG	
NORR01-F	GAGCTCAGGTTACCCGCATGATGTCCCAAACGATTGAC	
NORR01-R	GAAAGAGGGTAAATCCCACCATTTCTCC	Deletion of *norR* ORF
NORR02-F	GGTGGGATTTACCCTCTTTCCTGAACAAAC	
NORR02-R	CGACCCTCGAGTACGCGTCATTTTACGGCTGGGGTTTG	
**For site-directed mutagenesis**		
NSRRC91S-F	GGAGCCGTTGGATCTTGTCAATTCGTCGGTGGAGTTT	
NSRRC91S-R	AAACTCCACCGACGAATTGACAAGATCCAACGGCTCC	
NSRRC96S-F	TTGCTCGGTGGAGTTTTCGCATATTACGCCCGCATG	Construction of *nsrR*3CS mutant
NSRRC96S-R	CATGCGGGCGTAATATGCGAAAACTCCACCGAGCAA	
NSRRC102S-F	GCCATATTACGCCCGCATCGCGTTTGAAGGAGCG	
NSRRC102S-R	CGCTCCTCCAAACGCGATGCGGGCGTAATATGGC	
**For mutant complementation**		
NSRRC-F	GATCCCCGGGTACCAGCGGGCTTCCTATTAGGATTATT	Amplification of *nsrR* ORF
NSRRC-R	ACGAATTCGAGCTCTTAGGCTTTTTGCAGCAAGATCAG	
NSRREC01-F	CTCAGGTTACCCGCATGCGGCCACTATGTCGGTTTTAG	
NSRREC01-R	ATGAGCAACCCGACCTTTCCTTGGTGATC	Ectopic integration of *nsrR* regulatory region and ORF
NSRREC02-F	GGAAAGGTCGGGTTGCTCATGTTAAAGATATCTTTGAG	
NSRREC02-R	ATCACTTCCAAATGTGCGCCATGCCATTG	
NSRREC03-F	GGCGCACATTTGGAAGTGATCCCACAAC	

NSRREC03-R	CTCGAGTACGCGTCACTAGTCGGTCTTCTT CGTAGTGCACC	
**For qRT-PCR analysis**		
HMPA_qRT-F	GGCCGAAACAGGACCAAAAC	Quantification of *hmpA* expression
HMPA_qRT-R	TGCGAGTTTGACTTTGCTGC	
NNRS_qRT-F	AAATTCCCGCTTGGCTGAGA	Quantification of *nnrS* expression
NNRS_qRT-R	CAAAGGTAAGTTTGCCGCCC	
16S_qRT-F	CGGCAGCACAGAGAAACTTG	Quantification of 16S rRNA expression
16S_qRT-R	CCGTAGGCATCATGCGGTAT	
**For primer extension analysis**		
HMPAUP-F	TAATCAGTAAAGCTCGTCAGTTGC	Amplification of *hmpA* upstream region and extension of *hmpA* transcript
HMPAUP-R	TCCTGTTTCGGCCAACAGG	
NSRRUP-F	TAGCGGGCTTCCTATTAGGATTA	Amplification of *nsrR* upstream region and extension of *nsrR* transcript
NSRRUP-R	TATCCGGCAACGACGCGA	
**For protein overexpression**		
NSRRP-F	ACTTTAAGAAGGAGATATACCCATGGAATG CAACTGACGAGCTTTAC	Amplification of *nsrR* and *nsrR*$_{3CS}$ ORF
NSRRP-R	CAGTGGTGGTGGTGGTGGTGGGCTTTTTGC AGCAAGATC	
**For reporter construction**		
PnsrR-F	GAGCTCATTGCGTTGGTTGCTCATG	Amplification of *nsrR* regulatory region
PnsrR-R	ACTAGTGAAACACCAAATAGCTCAGTCAC AT	
**For EMSA and DNase I protection assay**		
PnsrRhmpA-F	GAAACACCAAATAGCTCAGTCACAT	Amplification of *nsrR-hmpA* regulatory region
PnsrRhmpA-R	ATTGCGTTGGTTGCTCATG	
Pisc-F	AATAAAATGCGTCGATTGTTCAG	Amplification of *isc* operon regulatory region
Pisc-R	CTCGGAAATATCAGCCAGAGG	

[a] The oligonucleotides were designed using the *V. vulnificus* MO6-24/O genomic sequence (GenBank[TM] accession number CP002469 and CP002470, www.ncbi.nlm.nih.gov).
[b] Regions of oligonucleotides not complementary to the corresponding genes are underlined.
