# Supplementary material for: A Nitric Oxide-Responsive Transcriptional Regulator NsrR Cooperates With Lrp and CRP to Tightly Control the hmpA Gene in Vibrio vulnificus
Source: Front Microbiol. 2021 May 21;12:681196. doi: 10.3389/fmicb.2021.681196 (PMC8175989; doi:10.3389/fmicb.2021.681196)
Supplement: Supplementary file 4 [file Image_1.pdf]

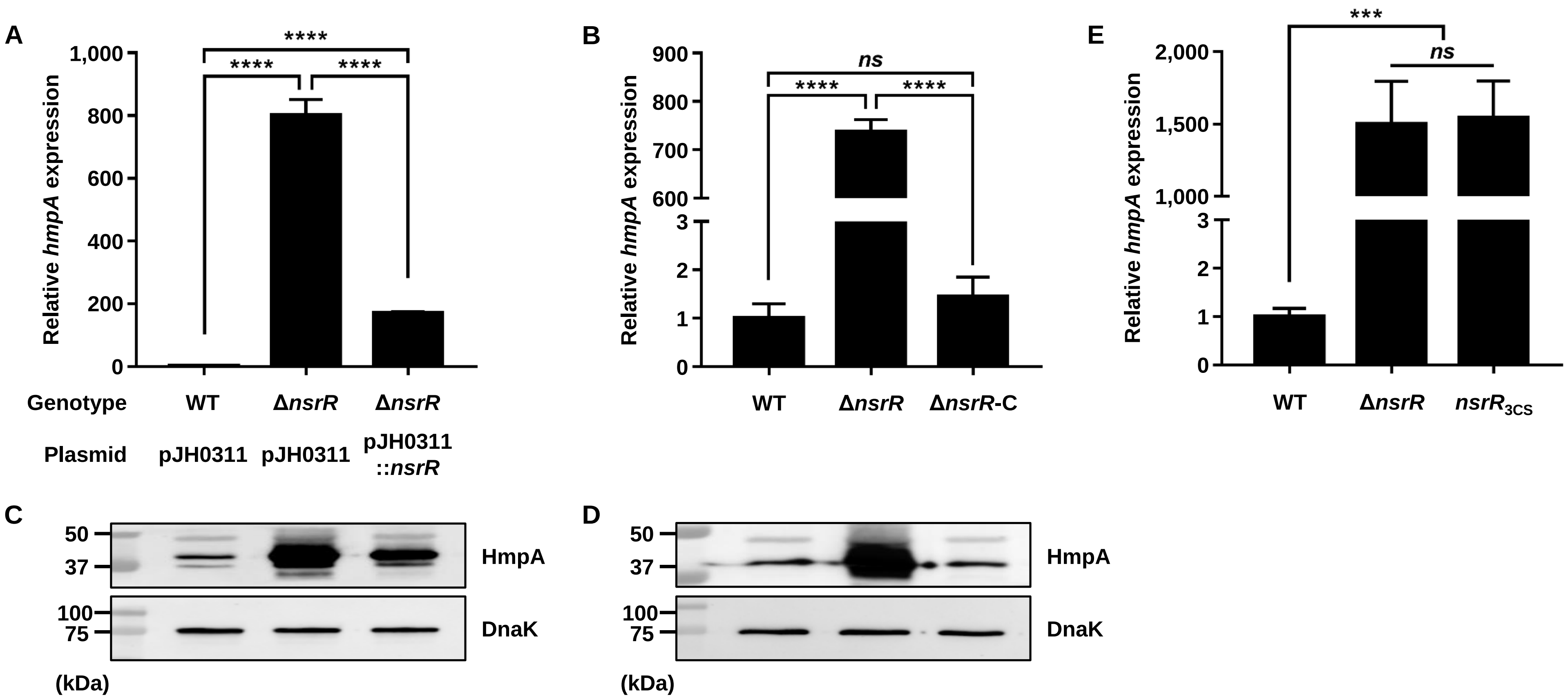

Supplementary Figure 1. The effect of the *nsrR* mutation on *hmpA* transcription. Total RNA and proteins were isolated from the wild-type and mutant strains grown aerobically to an A_{600} of 0.5. **(A,B,E)** The *hmpA* transcript levels were determined by qRT-PCR, and the *hmpA* transcript levels in the wild-type strain were set to 1. Error bars represent the SD. Statistical significance was determined by the Student's *t* test (***, $p < 0.0005$; ****, $p < 0.00005$; *ns*, not significant). **(C,D)** The cellular HmpA and DnaK (internal control) protein levels were determined by Western blot analysis. Molecular size markers (Bio-Rad) are shown in kDa. WT, wild type; Δ*nsrR*, *nsrR*-deletion mutant; pJH0311, broad-host-range vector; pJH0311::*nsrR*, pJH0311 carrying the *nsrR* gene (pDY1702); Δ*nsrR*-C, strain ectopically expressing NsrR; $nsrR_{3CS}$, strain expressing apo-locked NsrR.
