# Supplementary material for: A Nitric Oxide-Responsive Transcriptional Regulator NsrR Cooperates With Lrp and CRP to Tightly Control the hmpA Gene in Vibrio vulnificus
Source: Front Microbiol. 2021 May 21;12:681196. doi: 10.3389/fmicb.2021.681196 (PMC8175989; doi:10.3389/fmicb.2021.681196)
Supplement: Supplementary file 5 [file Image_2.pdf]

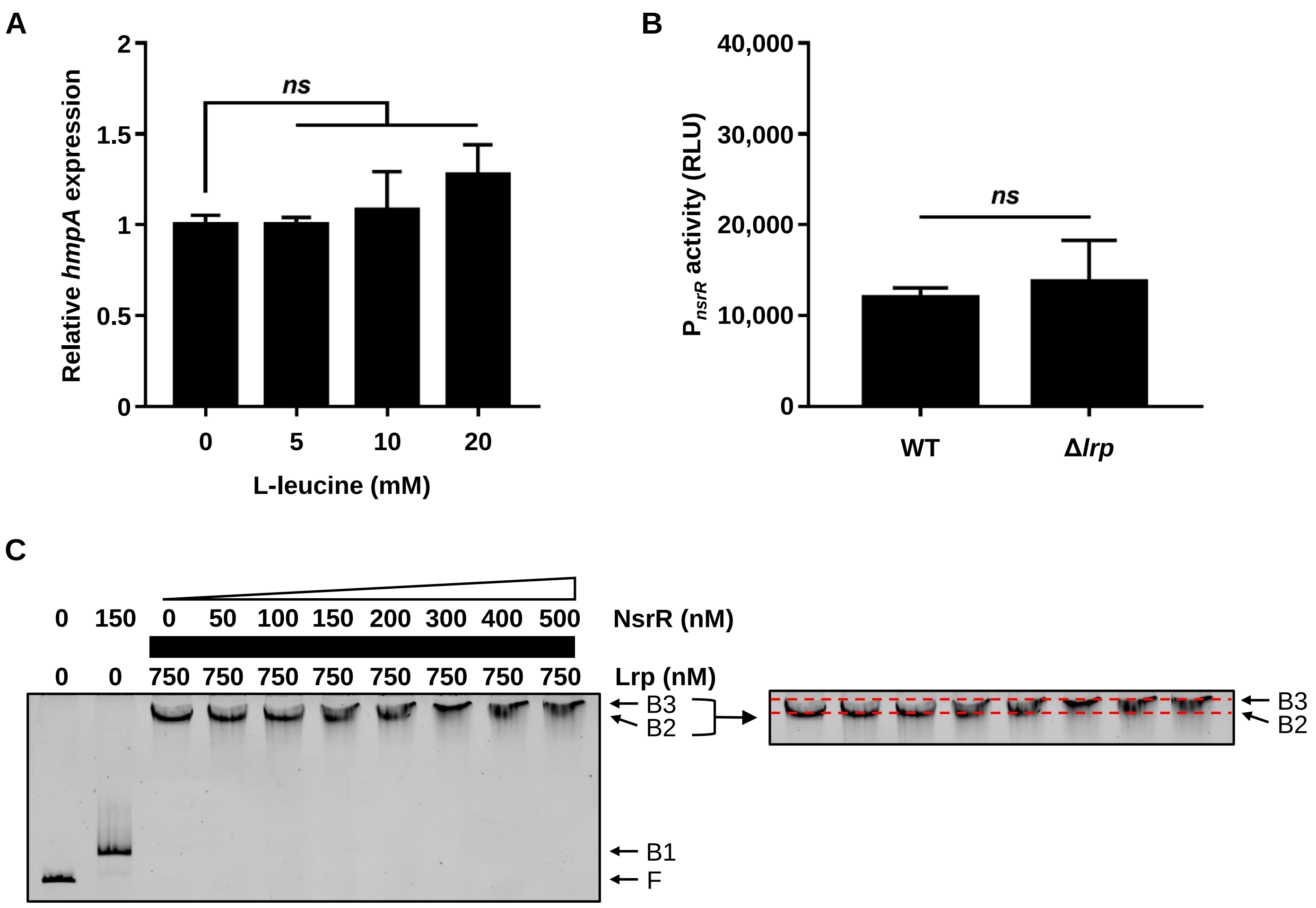

Supplementary Figure 2. The effect of leucine on *hmpA* transcription and interaction of NsrR and Lrp on the *nsrR-hmpA* regulatory region. **(A)** Total RNA was isolated from the wild-type strain grown aerobically to an A_{600} of 0.5 with or without various amounts of L-leucine. The *hmpA* transcript levels were determined by qRT-PCR, and the *hmpA* transcript level in the wild-type strain grown without L-leucine was set to 1. **(B)** A PCR fragment carrying the P_{nsrR} was cloned into pBBR-lux to create a reporter plasmid, pGR2025. The wild-type and Δ*lrp* strains containing pGR2025 were grown aerobically to an A_{600} of 0.5, and then used to measure the cellular luminescence. Error bars represent the SD. Statistical significance was determined by the Student's *t* test (*ns*, not significant). RLU, relative luminescence unit; WT, wild type; Δ*lrp*, *lrp*-deletion mutant. **(C)** A 393-bp DNA fragment of the *nsrR-hmpA* regulatory region (10 nM) was labeled with 6-FAM, and then incubated with increasing amounts of NsrR in the presence of 750 nM Lrp as indicated. B1, DNA-NsrR complex; B2, DNA-Lrp complex; B3, DNA-NsrR-Lrp complex; F, free DNA.
