# Supplementary material for: A Nitric Oxide-Responsive Transcriptional Regulator NsrR Cooperates With Lrp and CRP to Tightly Control the hmpA Gene in Vibrio vulnificus
Source: Front Microbiol. 2021 May 21;12:681196. doi: 10.3389/fmicb.2021.681196 (PMC8175989; doi:10.3389/fmicb.2021.681196)
Supplement: Supplementary file 6 [file Image_3.pdf]

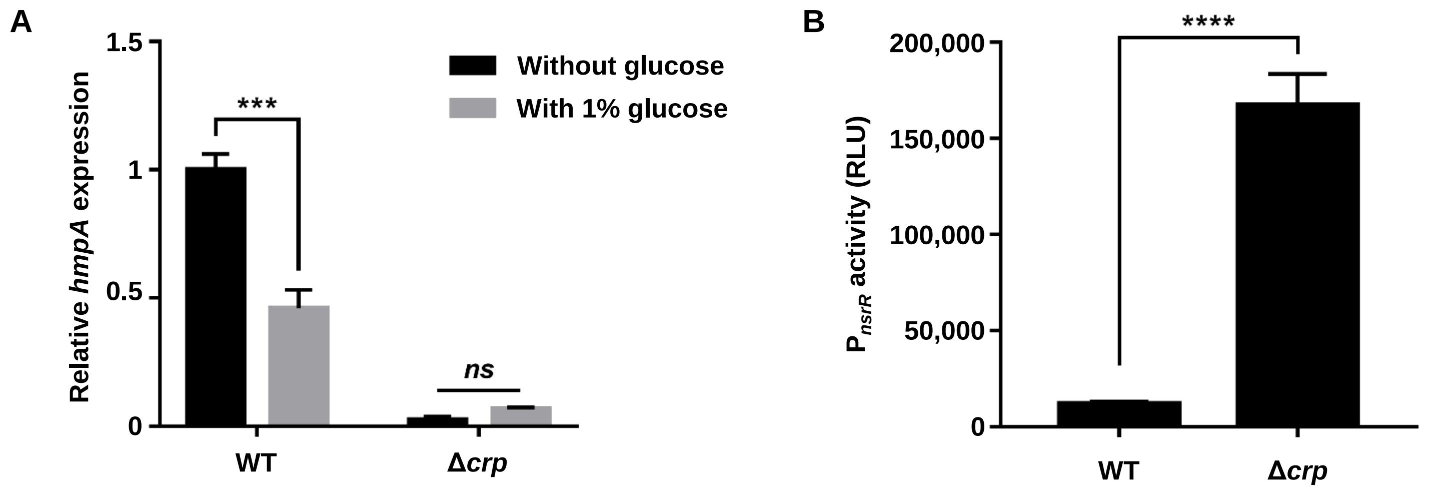

Supplementary Figure 3. The effect of glucose on *hmpA* transcription and the effect of the *crp* mutation on *nsrR* transcription. **(A)** Total RNA was isolated from the wild-type and Δ*crp* strains grown aerobically to an A_{600} of 0.5 with or without 1% glucose. The *hmpA* transcript levels were determined by qRT-PCR, and the *hmpA* transcript level in the wild-type strain grown without 1% glucose was set to 1. **(B)** A PCR fragment carrying the P_{nsrR} was cloned into pBBR-lux to create a reporter plasmid, pGR2025. The wild-type and Δ*crp* strains containing pGR2025 were grown aerobically to an A_{600} of 0.5, and then used to measure the cellular luminescence. Error bars represent the SD. Statistical significance was determined by the Student's *t* test (***, $p < 0.0005$; ****, $p < 0.00005$; *ns*, not significant). WT, wild type; Δ*crp*, *crp*-deletion mutant; RLU, relative luminescence unit.
