# Supplementary material for: A Nitric Oxide-Responsive Transcriptional Regulator NsrR Cooperates With Lrp and CRP to Tightly Control the hmpA Gene in Vibrio vulnificus
Source: Front Microbiol. 2021 May 21;12:681196. doi: 10.3389/fmicb.2021.681196 (PMC8175989; doi:10.3389/fmicb.2021.681196)
Supplement: Supplementary file 7 [file Image_4.pdf]

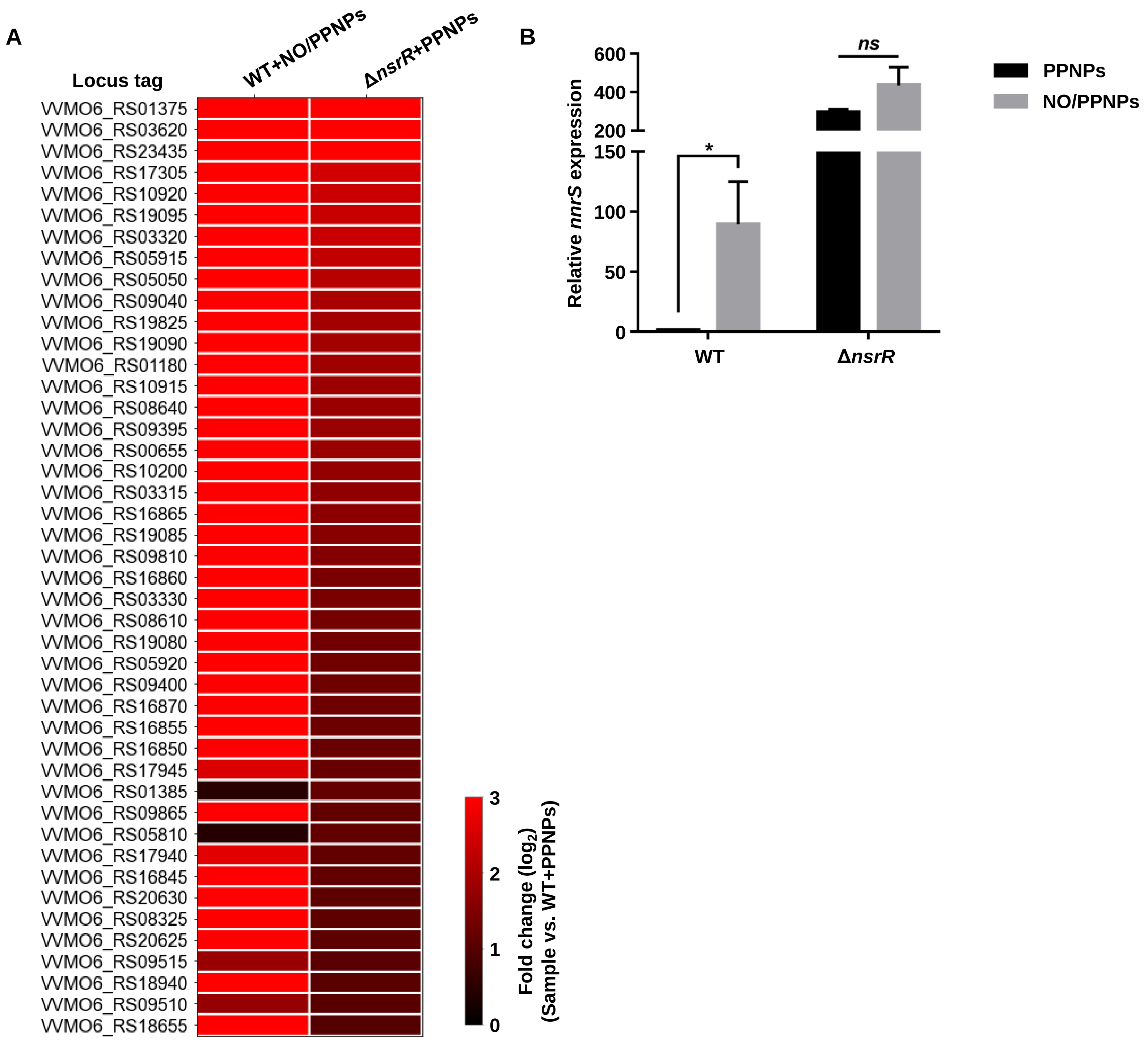

Supplementary Figure 4. The effect of nitrosative stress on NsrR regulon expression. **(A)** The expression of the 44 genes, up-regulated by the *nsrR* deletion, in either the NO/PPNPs-exposed wild-type (left panels) or PPNPs-exposed Δ*nsrR* (right panels) strain is presented relative to that in the PPNPs-exposed wild-type strain. The fold changes are visualized in the heat map with colors representing the $\log_2$ RPKM ratio. **(B)** To confirm the effect of NO and the *nsrR* mutation on *nnrS* transcription, the wild-type and Δ*nsrR* strains were grown aerobically to an A_{600} of 0.5 in M9G, and then exposed to 0.15 mg/ml PPNPs or NO/PPNPs for 10 min. The *nnrS* transcript levels were determined by qRT-PCR, and the *nnrS* transcript level in the wild-type strain exposed to PPNPs was set to 1. Error bars represent the SD. Statistical significance was determined by the Student's *t* test (*, $p < 0.05$; *ns*, not significant). WT, wild type; Δ*nsrR*, *nsrR*-deletion mutant.
