# Supplementary material for: A Nitric Oxide-Responsive Transcriptional Regulator NsrR Cooperates With Lrp and CRP to Tightly Control the hmpA Gene in Vibrio vulnificus
Source: Front Microbiol. 2021 May 21;12:681196. doi: 10.3389/fmicb.2021.681196 (PMC8175989; doi:10.3389/fmicb.2021.681196)
Supplement: Supplementary file 8 [file Image_5.pdf]

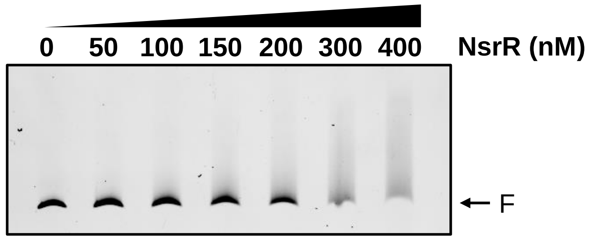

Supplementary Figure 5. Interaction between NsrR and the *isc* operon regulatory region. A 321-bp DNA fragment of the *isc* operon regulatory region (10 nM) was labeled with 6-FAM, and then incubated with increasing amounts of NsrR as indicated. F, free DNA.
