# Supplementary material for: A Nitric Oxide-Responsive Transcriptional Regulator NsrR Cooperates With Lrp and CRP to Tightly Control the hmpA Gene in Vibrio vulnificus
Source: Front Microbiol. 2021 May 21;12:681196. doi: 10.3389/fmicb.2021.681196 (PMC8175989; doi:10.3389/fmicb.2021.681196)
Supplement: Supplementary file 9 [file Image_6.pdf]

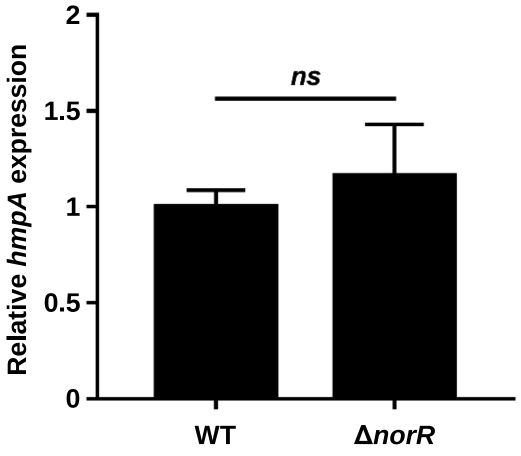

Supplementary Figure 6. The effect of the *norR* mutation on *hmpA* transcription. Total RNA was isolated from the wild-type strain and isogenic *norR*-deletion mutant grown aerobically to an A_{600} of 0.5. The *hmpA* transcript levels were determined by qRT-PCR, and the *hmpA* transcript level in the wild-type strain was set to 1. Error bars represent the SD. Statistical significance was determined by the Student's *t* test (*ns*, not significant). WT, wild type; Δ*norR*, *norR*-deletion mutant.
